# A small molecule 20C from *Gastrodia elata* inhibits α-synuclein aggregation and prevents progression of Parkinson’s disease

**DOI:** 10.1038/s41419-023-06116-0

**Published:** 2023-09-06

**Authors:** Ye Peng, Jun-rui Ye, Sha-sha Wang, Wen-bin He, Zhong-ping Feng, Hong-shuo Sun, Shi-feng Chu, Zhao Zhang, Nai-hong Chen

**Affiliations:** 1 School of Pharmacy, Minzu University of China, Beijing, 100081 China; 2 State Key Laboratory of Bioactive Substances and Functions of Natural Medicines, Institute of Materia Medical & Neuroscience Center, Chinese Academy of Medical Sciences and Peking Union Medical College, Beijing, 100050 China; 3 Science and Technology Innovation Center, Guangzhou University of Chinese Medicine, Guangzhou, 510405 China; 4 Shanxi University of Chinese Medicine, National International Joint Research Center for Molecular Chinese Medicine, Taiyuan, 030024 China; 5 Department of Physiology, Faculty of Medicine, University of Toronto, Toronto, ON M5S 1A8 Canada

**Keywords:** Drug development, Parkinson's disease

## Abstract

Parkinson’s disease (PD) is pathologically manifested by the aggregation of α-synuclein, which has been envisioned as a promising disease-modifying target for PD. Here, we identified 20C, a bibenzyl compound derived from *Gastrodia elata*, able to inhibit the aggregation of A53T variants of α-synuclein directly in vitro. Computational analysis revealed that 20C binds to cavities in mature α-synuclein fibrils, and it indeed displays a strong interaction with α-synuclein and reduced their β-sheet structure by microscale thermophoresis and circular dichroism, respectively. Moreover, incubating neural cells with 20C reduced the amounts of α-synuclein inclusions significantly. The treatment of A53T α-Syn transgenic mice with 20C significantly reduces the toxic α-synuclein levels, improves behavioral performance, rescues dopaminergic neuron, and enhances functional connections between SNc and PD associated brain areas. The transcriptome analysis of SNc demonstrated that 20C improves mitochondrial dynamics, which protects mitochondrial morphology and function against α-synuclein induced degeneration. Overall, 20C appears to be a promising candidate for the treatment of PD.

## Introduction

Parkinson’s disease (PD) is the second most common neurodegenerative disorder among middle-aged and elderly people, after Alzheimer’s [1]. However, available drugs could only improve motor dysfunction, but failed to block the progression of its pathology [2, 3], which could not relieve the burdens induced by PD.

The principle pathological feature of PD is the loss of dopaminergic neurons, accompanied by the accumulation of misfolded α-synuclein (α-Syn) in Lewy bodies and Lewy neurites [4, 5]. In-depth autopsy neuropathological examination of patients with PD and related synuclein disease revealed different subtypes of pathological inclusions that were enriched in aggregated α-Syn, including fibrils [6, 7]. α-Syn is distributed in neurons as a disordered protein that participates in neurotransmitter release [8], whereas in its pathological state, α-Syn misfolds and aggregates to form oligomers or amyloid fibrils, which further promote mitochondrial dysfunction, oxidative stress injury, endoplasmic reticulum stress, neuroinflammation, and damage axonal transport [8].

Mitochondrial dysfunction maybe one of core synucleinopathy in PD [9–11]. A study of mitochondrial α-Syn in the human brain demonstrated that α-Syn was significantly accumulated in the mitochondria of patients with PD’s substantia nigra and striatum [12]. Long-term exposure to environmental toxins is a known risk factor for the development of PD, and many of these compounds are toxic primarily at mitochondrial level. The administration of mitochondrial poisons (such as MPTP and paraquat) to rodents and cell cultures resulted in the formation of α-Syn aggregates and inclusions [13]. Similarly, inhibiting the ubiquitin proteasome pathway in vitro causes unfolded protein accumulation, mitochondrial dysfunction, and neuronal cell death [14]. α-Syn has been shown in previous studies to promote mitochondrial division and inhibit fusion by activating Mfn1, Mfn2, and OPA1 [15]. The role of α-Syn aggregation and mitochondrial dysfunction in the pathogenesis of PD has been thoroughly established. These two processes are intertwined and mutually beneficial in pathogenesis [16].

*Gastrodia elata*, as a traditional Chinese medicine, has been used to treat headache, dizziness, epilepsy, tremor and paralysis from 2000 years ago, which are the principal symptoms of PD. The novel bibenzyl compound 20C, present in *Gastrodia elata*, exhibited a significant enhancement in cell viability of the PD model [17]. In addition, it mitigated motor dysfunction and prolonged the progression of pathological changes in vivo [18]. These findings indicated that 20C may serve as a crucial bioactive component for the treatment of PD in *Gastrodia elata*. However, the drug target and underlying mechanism are still obscure. Here, we tested the interaction between 20C and synuclein and investigated its inhibitory activities in the aggregation processing of A53T α-Syn. Finally, we validated the effects using a model of A53T α-Syn transgenic mice. These findings provided a theoretical foundation for drug research and clinical transformation of 20C.

## Results

### 20C inhibits A53T α-Syn aggregation in vitro

20C, a novel bibenzyl compound, has been identified as one of the molecules of potential interest by anti-PD compound screening isolated from *Gastrodia elata* (Fig. S1A–D). However, the drug target of 20C is still unclear. We used a multidisciplinary approach in this study to investigate the effect of 20C on the aggregation process and products of A53T α-Syn. The A53T mutation increased fibrils proliferation and toxicity in vitro and exacerbated PD-like pathological features in cells and animal models [19], but the mutation has no effect on the natural unfolded or partially folded intermediate conformation of α-Syn [20–23].

**Fig. 1:**
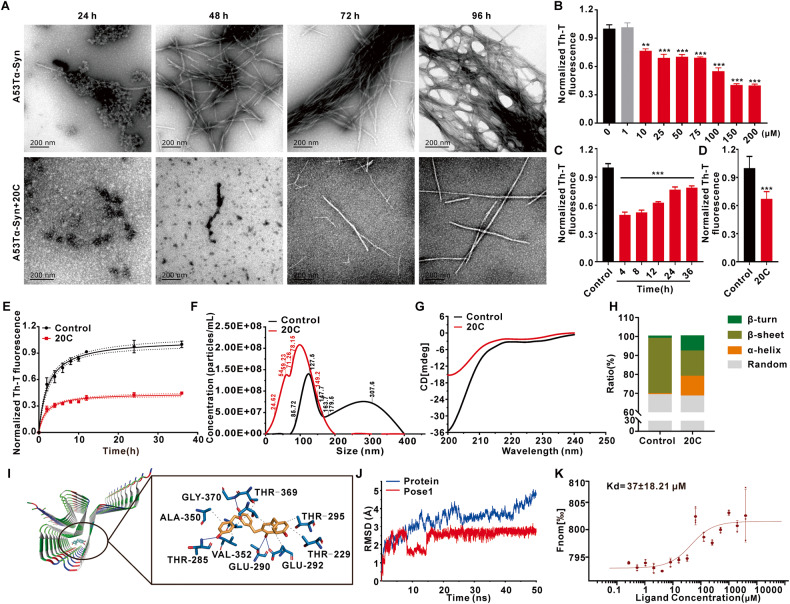
20C inhibits A53T α-Syn aggregation in vitro. **A** Representative TEM images in absence (upper) and presence (bottom) of 20C (100 μM) at different time. **B** Inhibition of A53T α-Syn aggregation by 20C revealed by Th-T fluorescence from 1 to 200 μM after incubation of 96 h. Normalized to the group of 0 μM. **C** Inhibitory effect of 20C (100 μM) on the maturation of α-syn aggregates from different states. 20C was dropped into the aggregation solution after 4, 8, 12, 24, 36 h of incubation, and their aggregation mature status was determined at the time of 96 h. **D** The end point (96 h) of A53T α-Syn with or without (control) of 20C (100 μM). **E** A53T α-Syn aggregation kinetics in the absence (control) and presence of 20C followed by Th-T derived fluorescence. Normalized to the end point Th-T fluorescence of the control group at the time of 36 h. **F** Determination of the fibril size population in absence (black) and presence (red) of 20C using nano ZS system. **G** Representative image of CD spectroscopy for the secondary structure of full-length α-Syn in absence (black) and presence (red) of 20C. **H** α-Syn secondary structure contents affected by 20C. **I** The binding mode based on 20C-fibril interaction dynamics simulation, overall view (left) and local view (right). The yellow rod-like structure is 20C, the blue rod-like structure is a protein residue, the blue solid line represents hydrogen bonding, and the gray dotted line represents hydrophobicity. **J** RMSD results of 20C-fibril complex with time in the simulation process. **K** Dose–response curve of 20C and α-Syn fibrils interaction determined by MST. Error bars are represented as SEM of mean values, *n* = 3. **p < 0.01, ***p < 0.001 vs. the control group.

The inhibitory of 20C on the aggregation kinetics of A53T α-Syn was revealed by transmission electron microscope (TEM) and thioflavin-T (Th-T) analysis, respectively. As shown in Fig. 1A, there was significant fibril formation in the control group after 48 h of incubation, which converted to black layers at 72 h and fibril clusters at 96 h. However, the fibril in 20C group was not found until 72 h, and there are no black layers or fibril clusters until 96 h. Th-T fluorescence demonstrated that 20C inhibited A53T α-Syn aggregation in a concentration-dependent manner from 10 μM (20C: α-Syn=1:7) and reaching its peak at 150 μM. (Fig. 1B). Next, we investigated the effect of 20C on the fibril clusters mature from different time of α-Syn aggregation. The results revealed that 20C effectively inhibited the further maturation of α-Syn at various times (Fig. 1C, D). The polymerization curve revealed that the Th-T fluorescence intensity in 20C group was significantly lower than that of control group after 36 h, indicating that α-Syn aggregation was reduced in the presence of 20C, and the reduction was more than 50% at the end of this study (Fig. 1E). All these findings suggested that 20C could inhibit α-Syn aggregation into fibril clusters. Moreover, the diameter of α-Syn aggregation product was tested by nano-particle size detection. As shown in Fig. 1F, the diameter of control aggregates is greater than 200 nm, while the presence of 20C significantly reduces the production of large-sized protein aggregates, manifested by the increased number of <100 nm nanoparticles and decreased number of >200 nm aggregates. To examine whether this range of α-Syn in the 20C group could induce neuronal damage, we observed SH-SY5Y cells. As shown in Fig. S1E–I, compared to the control group, neuronal damage induced by synuclein oligomers in the 20C group was significantly reduced, and the cell incubation significantly decreased the levels of synuclein oligomers in the cell culture medium. The results of particle size detection showed that after treatment with proteinase K, the protein particle size in the 20C group significantly decreased and remained below 10 nm, while the control group (without 20C) maintained large particles that were resistant to proteinase K digestion, with protein particle size concentrated around 300 nm. After proteinase K treatment, the low molecular weight proteins significantly increased, and the high molecular weight proteins significantly decreased in the 20C group. At the same time, the addition of the autophagy inhibitor 3-Methyladenine (3-MA) and the proteasome inhibitor MG132 did not prevent the significant reduction of synuclein aggregates mediated by 20C. Based on these results, although the diameter of synuclein aggregates in the 20C group reaches the toxic range, their low stability prevents them from inducing cellular toxicity.

The primary mechanism underlying α-Syn aggregation is the change in their secondary structure, which is embodied in the transition from α-helix to β-sheet. Circular dichroism (CD) determination showed that application of 20C altered the secondary structure spectrum of α-Syn significantly (Fig. 1G). α-helix structure in the control group accounts for 0.5%, while it increased to 10.4% in the 20C group. Moreover, β-sheet structure in the control group’s accounts for 29.2%, who decreased to 13.3% in the 20C group (Fig. 1H). These findings imply that 20C directly inhibits the aggregation of α-Syn.

### α-Syn fibrils can accommodate 20C

To validate our prediction, we used computer-aided technology to create a possible binding mode of α-Syn-20C and used a simplified model of α-Syn fibrils. The full-length α-Syn chain is replaced by 34–99 residues in the center, while the toxic structure of α-Syn is preserved, and this model was used as an effective replacement for the full-length α-Syn fibrils in all calculations [24]. We found three major interactions with α-Syn fibrils (Fig. S2A). These are the positions where 20C is most likely to bind into the β-sheet structure of fibrils, and they are all completely inserted into the fibril structure. The simulations of the three poses are performed using molecular dynamics (MD). The root mean square deviation (RMSD) (Fig. 1J, Fig. S2B) showed that pose 1 is much more stable after 20 ns than the other two poses, and the fluctuation is almost controlled in a straight line of 2.5 Å. Pose 1 had a lower root mean square fluctuation (RMSF) and formed significantly more hydrogen bonds indicated that it has less structural flexibility and more stability than the other two poses (Fig. S2C, D). Supplementary Table S1 showed the binding free energies of the three poses, which were −37.49, −28.23, and −26.85, respectively. To summarize, the ligand was sandwiched between two parallel β-sheets of the Greek-key motif and forms hydrogen bonds with the side chains of fibrils of THR-285, GLY-370, THR-290, and GLU-292, as well as hydrophobic interactions with ALA-350, THR-369, THR-295, THR-229, GLU-292, and VAL-352 (Fig. 1I). To further understand the interaction, we performed molecular dynamics binding simulations using a β-Synuclein variant lacking eight hydrophobic amino acids extending from residues 61–95 in the NAC domain. Results indicated that the binding between 20C and β-Synuclein was highly unstable, with continuous large fluctuations in rmsd observed throughout the 50 ns dynamic simulation period (Fig. S2E–G). Based on these calculations, 20C was predicted to bind into the core of synuclein fibrils.

Microscale thermophoresis (MST) was used to determine whether there is a direct interaction between α-Syn fibrils and 20C. MST is a technique for studying the interactions of biomolecules in an aqueous environment with no special solvent requirements. During our experiments, we used the fluorescence probe RED-NHS to label α-Syn fibrils by amino coupling, and we recorded the time-dependent fluorescence intensity curve (Fig. S3A, C, E) as different ligands interacted with α-Syn fibrils. Firstly, we incubated α-Syn antibody with protein as the positive control to confirm the fluorescence-labeled protein, and the Kd value of α-Syn antibody to protein was 2.3 ± 0.83 nM (2.3E-09 ± 0.83 M) (Fig. S3B). The results showed that the system could be used to determine the interaction between small molecules and α-Syn fibrils. A combined binding curve was plotted using various concentrations of 20C (highest concentration 4.10 mM) incubated with α-Syn fibrils. The Kd value was calculated to be 37 ± 18.21 μM (3.7E-05 ± 1.82 M) (Fig. 1K). The solvent control experiment that when the solvent was added alone and tested for interaction with α-Syn, the binding curve could not be fitted (Fig. S3D). All these findings indicated that there is a direct interaction between 20C and α-Syn.

### 20C inhibits the formation of α-Syn aggregates in SNCA (A53T) overexpression neurons

**Fig. 2:**
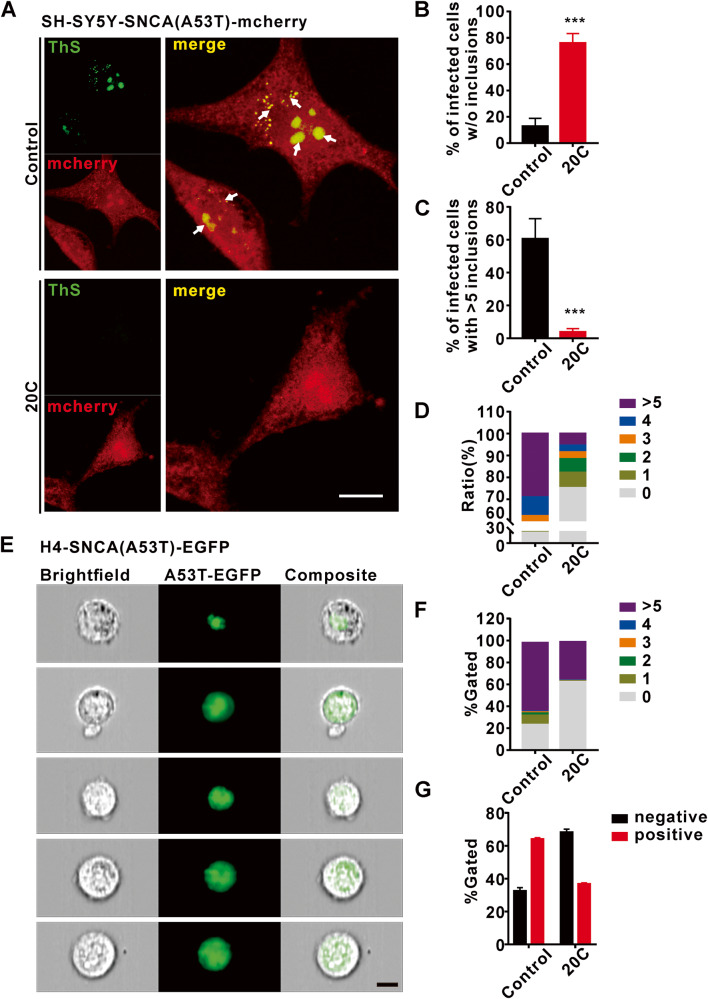
20C inhibited of α-Syn aggregate in SNCA (A53T) overexpression neurons. **A** Representative immunofluorescence images from SNCA (A53T)-SY5Y neurons treated with (bottom) or without 20C (Top). Scale bar = 10 µm. **B** Percentage of infected cells devoid of α-Syn aggregates. **C** Percentage of infected cells bearing >5 α-Syn aggregates. **D** Percentage of infected cells of infected SNCA (A53T)-SY5Y containing different α-Syn aggregates. **E** Representative immunofluorescence images of EGFP-positive cells revealed by imaging flow cytometry. Images of positive cells with 1, 2, 3, 4, >5 α-Syn aggregates are represented from top to bottom. **F** Percentage of infected cells of infected SNCA (A53T)-H4 containing different α-Syn aggregates. **G** Percentage of infected positive cells. Error bars are represented as SEM of mean values, *n* = 3. ***p < 0.001 *vs*. the control group.

In light of the interaction between 20C and α-Syn, we observed the effect of 20C on intracellular synuclein aggregation. We assessed α-Syn inclusion formation using a well-established cell model. SH-SY5Y cells were infected with the SNCA (A53T)-mcherry lentivirus. H4 cells were transiently transfected with the SNCA (A53T)-EGFP fusion virus. Immunofluorescence (Fig. 2A) and imaging flow cytometry were used to assess the formation of α-Syn inclusions 24 h after treatment with 20C (Fig. 2E, S5). Upon treatment with 10 μM 20C, we observed a significant increase in the number of transfected cells devoid of α-Syn inclusions (for SH-SY5Y, 20C: 75.95 ± 7.3%; for H4, 20C: 62.27 ± 1.13%) relative to untreated samples (for SH-SY5Y, control: 12.75 ± 6.01%; for H4, control: 24.52 ± 5.48%) (Fig. 2B, F). Th-T staining was used to detect the formation of α-Syn aggregates. 20C significantly reduced the proportion of cells with more than five aggregates (for SH-SY5Y, 20C: 3.91 ± 2.07%; for H4, 20C: 35.15 ± 1.62%) relative to the control group (for SH-SY5Y, control: 60.42 ± 12.35%; for H4, control: 63.14 ± 5.78%) in positive cells (Fig. 2C, F). The classified statistics of the number of aggregates formed in positive cells infected with SNCA (A53T) in the entire system, as shown in Fig. 2D for SH-SY5Y and Fig. 2G for H4, show that 20C effectively reduces the production of α-Syn aggregates in A53T overexpression neurons. In order to exclude false positives caused by changes in cell activity caused by 20C, we tested the potential toxicity to 20C to SNCA (A53T) overexpression neurons, the result showed that 20C was innocuous at concentrations as high as 50 μM (Fig. S4). All of which indicated that 20C inhibited the formation of α-Syn aggregates.

### 20C improves the behavioral performance in A53T α-Syn transgenic mice

**Fig. 3:**
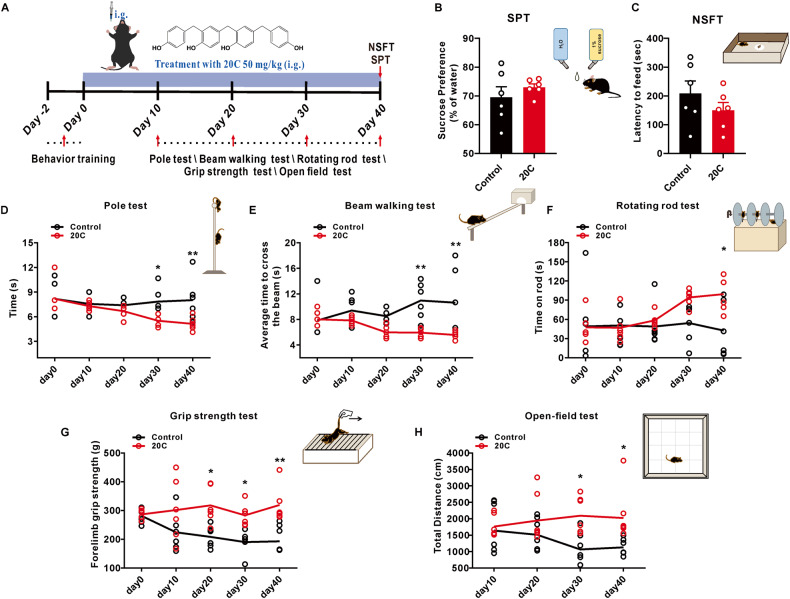
20C improved behavioral performance in α-Syn A53T transgenic mice. **A** Diagram of the experimental design. 20C (50 mg/kg) or vehicle (0.5% CMC-Na) was intragastric (i.g.) for 40 consecutive days, their motor behavioral tests were performed on day 10, 20, 30 and 40, and emotional states were tested after the last motor determination, then, tissues were harvested for follow-up analyses. **B** The sucrose preference in the sucrose preference test. **C** The latency to feed in the Novelty suppressed feeding test. Time to descend pole (**D**), to traverse beam apparatus (**E**), to fall latency from an accelerating rotarod (**F**) and grip strength analysis (**G**). **H** Total distance of open-field test. Error bars are represented as SEM of mean values, *n* = 6. *p < 0.05, **p < 0.01 *vs*. the control group.

Our previous research found that the optimal dosage of 20C was 50 mg/kg for the treatment of PD [25]. In this study, an oral administration dose of 50 mg/kg was used to evaluate its anti-PD efficacy in A53T α-Syn transgenic mice. As shown in Fig. 3A, behavioral test array was introduced to evaluate the improvement of behavioral performance by 20C.

To exclude the influence of mood changes on their behavioral outcomes, novelty suppressed feeding test and sucrose preference test were introduced. As shown in Fig. 3Β, C, long term administration of 20C did not change the latency to feed and the percent of sucrose consumption, indicated that their emotional state did not affect subsequent motor function tests.

Motor dysfunction is the principal symptom of PD, which was tested by rotating rod test, pole test and beam walking task. It was found there is a significant improvement on their motor dysfunction after treatment of 30 days, manifested by the reduced latency of mice climbing down in the pole test (Fig. 3D), shortened the cross time in the beam walking test (Fig. 3E), and increased the latency of fall down from the rotating rod (Fig. 3F) with the prolongation of 20C treatment. Simultaneously, the forelimb grip of mice in the 20C group was significantly enhanced (Fig. 3G), In the open-field test, we found that the total exercise distance was significantly increased in the 20C group (Fig. 3H), both of which are helpful for their performance in these tests. In addition, we administered the drug at 20C to wild-type mice of the same age, ruling out the possibility that the behavioral improvement observed was due to excessive stimulation caused by 20C (Fig. S6A–E). Moreover, we conducted neuronal observations in SNc at both 30 and 40 days of treatment. The results demonstrated that the 20C group exhibited a neuroprotective effect after 30 days of treatment which was consistent with the duration of behavioral improvement (Fig. S6F, G). The findings suggest that 20C improves motor disturbance and motor coordination in PD transgenic mice.

### 20C improves brain functional connection in A53T α-Syn transgenic mice

**Fig. 4:**
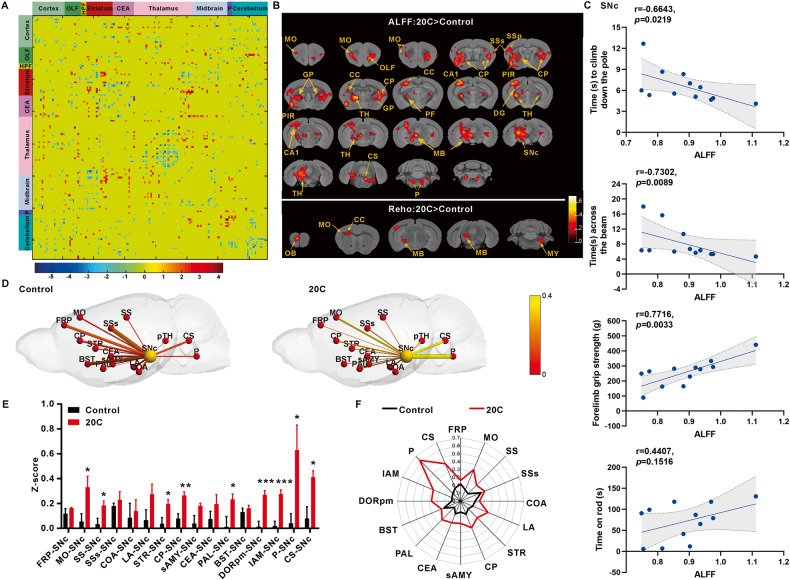
20C improved the functional connectivity in α-Syn A53T transgenic mice. **A** Matrix representation of the whole-brain functional connectome (151 ×151 nodes) in the mouse brain, obtained from the T-value of two-sample t-test. **B** Statistical results of ALFF and ReHo in 20C group in comparison with that in control group. The voxel-level height threshold of ALFF was *p* < 0.01 (uncorrected) and the cluster-extent threshold were 20 voxels. The voxel-level height threshold of ReHo was *p* < 0.01 (uncorrected) and the cluster-extent threshold were 10 voxels. Brain regions with significant differences are indicated by different colors according to T-values. HPF Hippocampal formation, CEA Central amygdalar nucleus, MO Motor cortex, SSs Supplemental somatosensory area, SSp Primary somatosensory area, GP Globus pallidus, PIR Piriform area, CC Corpus callosum, TH Thalamus, CP Caudoputamen, PF Parafascicular nucleus, CA1 Field CA1, DG Dentate gyrus, MB Midbrain, SNc The compact part of substantia nigra, P Pons, OB Olfactory bulb, MY Medulla. **C** The relationship between the assessed motor behaviors and the ALFF changes in SNc induced by A53T and 20C. **D** The functional connectivity between pairs of nodes is marked by lines of increasing thickness with increasing Z-score values, indicating increasing functional connectivity. The color size of the nodes distinguishes the SNc from other brain regions. **E** Z-score values histogram of connections between SNc and other brain regions. **F** Z-score values of connections of SNc to other brain regions used a radar map. Error bars are represented as SEM of mean values, *n* = 3. *p < 0.05, **p < 0.01, ***p < 0.001 *vs*. control group.

We scanned the brain of A53T α-Syn transgenic mice that had been treated with 20C for 40 days. Transgenic mice’s entire brain were divided into 151 brain regions, including the prefrontal cortex, motor cortex, striatum, caudate putamen, globus pallidus, substantia nigra pars compacta, thalamus, and pons. As shown in Fig. 4A, the functional connection (FC) strength between each two regions were quantified, and the connection strength of between two groups of samples was analyzed using the t-test. On the whole brain FC, there was an increase of 211 significant FCs. The findings revealed a significantly enhanced connection between the striatum, midbrain, and cerebellum.

We compared the regional aberrant amplitude low-frequency fluctuation (ALFF) and regional homogeneity (ReHo) in the 20C group to that in the control group to determine the global effects of transgenic mice brain activity. ALFF stands for the amplitude of regional spontaneous neuronal activity, whereas ReHo measures coordination in regional neural activity. The uncorrected voxel-level height threshold for ALFF was *p* < 0.01 (uncorrected) and the cluster-extent threshold was 20 voxels. The increased ALFF level of 20C group focus on the nigrostriatal system and the extended brain stem (midbrain-pons-thalamus) in transgenic mice brain regions included motor-sensory cortex, caudate putamen, globus pallidus, striatum, hippocampus, thalamus, substantia nigra-midbrain, and pons. The voxel-level height threshold was *p* < 0.01 (uncorrected) in ReHo analysis, and the cluster-extent threshold was 10 voxels. 20C increased ReHo levels in the olfactory bulb, motor area, fornix, midbrain, and medulla oblongata of transgenic mice (Fig. 4B). In addition, correlation analysis was performed between the ALFF and ReHo changes in these brain regions and the behavioral outcomes related to motor function. As shown in Fig. 4C and Fig. S7A, B, the results revealed significant correlations between the ALFF values of the primary regulatory motor pathway involved in PD, i.e., the motor cortex-striatum-substantia nigra pathway and the motor behavior of the mice. Notably, the ALFF value of the motor cortex (MO) showed a negative correlation with the time taken by the mice to climb down the pole (*p* = 0.0278). The ALFF value of the caudoputamen (CP) exhibited a positive correlation with the duration of mouse stay on the rotating rod experiment (*p* = 0.0458). In addition, the ALFF value of the substantia nigra (SNc) demonstrated a negative correlation with the time taken by the mice to climb down the pole (*p* = 0.0219), a negative correlation with cross the balance beam (*p* = 0.0089), and a positive correlation with grip strength (*p* = 0.0033).

The brain regions of interest (ROI) were chosen for further investigation. There was a statistically significant difference between the 20C and control groups. We studied the FC matrix composed of the motor connection transmission region [26], the dopaminergic innervation region [27–29], the autonomic neural network information transmission region [30], the movement-respiratory scrolling correlation region [31], and the cognitive related information transmission region [32, 33] were compared with that of the control group (Fig. S7C). The connection strength of the matrix of transgenic animals in the 20C group was significantly increased throughout the network, and the overall color changed to yellow, indicating that the Z-score value of the 20C group was significantly increased. The FC strength of 20C group was greater than that of the control group among the ROI connections selected above (Fig. S7D). Similarly, the t-test revealed that functional network in the 20C group was enhanced significantly (Fig. S7E).

The compact part of substantia nigra (SNc) is particularly sensitive to PD, and it is also one of the key areas affected by α-Syn. As a result, we paid special attention to changes in the strength of the connection between SNc and PD related other regions. The connections between SNc and many brain regions were strengthened after 20C administration, particularly in brain regions associated with the onset and progression of PD. The connection between SNc and motor cortex (MO), somatosensory area (SS), striatum (Str), caudoputamen (CP), and globus pallidus (GP) was enhanced in the 20C group in the cortical-striatal connection network related to PD motor function. In the mesencephalic limbic-striatal ring connection network, the connection between SNc and part of thalamus (pTH) also increased. In addition, the connection between SNc and extended brain stem (midbrain-pons-thalamus) also increased significantly in the 20C group. In the regulatory network of cognitive and emotional disorders associated with PD symptoms, the connection between SNc and cortical amygdalar area (COA), frontal pole (FRP) and bed nuclei of the stria terminalis (BST) showed an enhanced trend in the 20C group. The above results showed that the connections between SNc and motor sensory cortex, caudate putamen and pons-thalamus in the 20C group were significantly enhanced.

The functional strength of internal connections in each ROI was reflected in additional intra-node connections analysis. Different linewidths represented FC enhancement between nodes, and spheres of various sizes and colors are used to distinguish the SNc from other brain regions. Figure 4D showed that the connection strength between different nodes and SNc nodes was significantly stronger in the 20C group than in the control group. The mean of the functional connection’s Z-score value clearly showed the enhancement of the node connection strength in the 20C group via the histogram (Fig. 4E) and the radar chart (Fig. 4F). These findings provide another evidence from neuroimaging for the improvement of behavioral performance induced by 20C.

### Protective effect of 20C on substantia nigra-striatum system

**Fig. 5:**
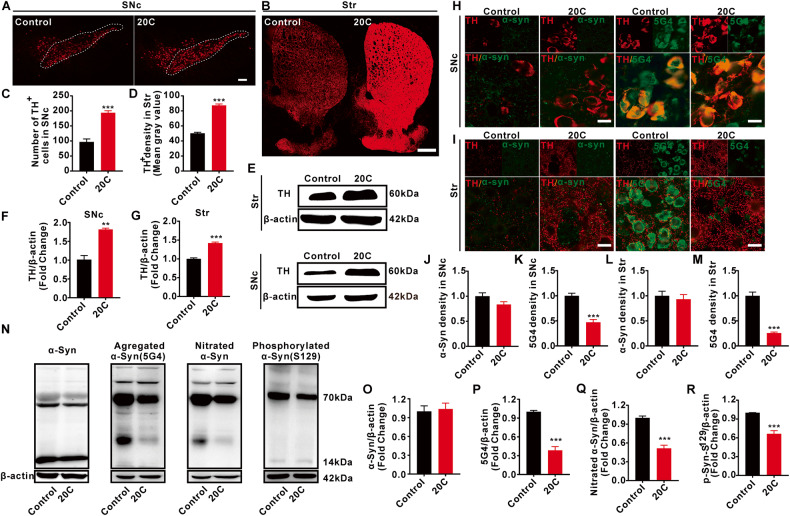
Protective effect of 20C on the nigrostriatal system. **A** 20C inhibited the loss of DA neurons in SNc revealed by immunofluorescence. Scale bar = 100 µm. **B** 20C reduced the loss of DA fibers in striatum (Str). Scale bar = 500 µm. **C** Quantification analysis of DA neurons in SNc. The cell count for the SNc was obtained using a dot counting method to determine the number of positive neurons. **D** Quantification analysis of DA fibers in Str. The mean optical density value of TH-positive staining in Str was quantified using Fuji software. **E** Representative immunoblot of TH protein levels in SNc and Str. **F**, **G** Quantification analysis of TH protein levels in SNc or Str. **H**, **I** Representative immunofluorescence images of the levels of α-Syn or aggregated α-Syn (5G4) in TH-positive neurons. Scale bar=20 µm. **J** Quantification analysis of α-Syn density in SNc. **K** Quantification analysis of 5G4 density in SNc. **L** Quantification analysis of α-Syn density in Str. **M** Quantification analysis of 5G4 density in Str. **N** Representative immunoblot of α-Syn and its modified forms in SNc. **O**–**R** Quantification analysis of (**N**). Normalized to the control group. Error bars are represented as SEM of mean values, *n* = 3. **p < 0.01, ***p < 0.001 *vs*. control group.

Progressive loss of dopaminergic (DA) neurons in the SNc is one of the main pathological features of PD. 20C protected against the loss of DA neurons (Fig. 5A, C; Fig. S8A) and the reduction in tyrosine hydroxylase (TH) immunoreactivity in SNc and Str (Fig. 5E–G). 20C led to a greater TH^+^ fibrils density in Str in comparison to control (Fig. 5B, D). The transmission electron microscopic observation of neuronal morphology showed that 20C could improve the shrinkage of neuronal nucleus in A53T transgenic mice (Fig. S9).

Another pathological feature of PD is abnormal aggregation of α-Syn into Lewy bodies. Results in figure S8B demonstrated that 20C treatment significantly reduces the content of Lewy bodies in SNc. 20C protected against the fluorescence expression of aggregated α-Syn in SNc and Str (Fig. 5H, I, K, M), relieved the increased in aggregated α-Syn (Fig. 5N, P) and nitrified α-Syn immunoreactivity in SNc (Fig. 5N, Q). Aggregated synuclein is usually accompanied by abnormal phosphorylation, with the most prominent modification occurring at serine 129, 20C significantly reduces the phosphorylation S129 of synuclein in SNc (Fig. 5N, R). About the total α-Syn, there was no significant difference in the expression of monomer α-Syn protein with molecular weight of 15 kDa (Fig. 5N, O) and no significant difference in the fluorescence expression of α-Syn in SNc and Str between the two groups (Fig. 5H–J, L), but there was a difference in the expression of α-Syn protein at about 70 kDa with higher molecular weight (no statistics). These results suggested that 20C effectively protected the DA neurons and improved pathological synuclein by reduced the abnormal modification of α-Syn in the nigra-striatum of A53T α-Syn transgenic mice.

### Mitochondrial organization were identified as the primary biological processing of 20C to against PD

**Fig. 6:**
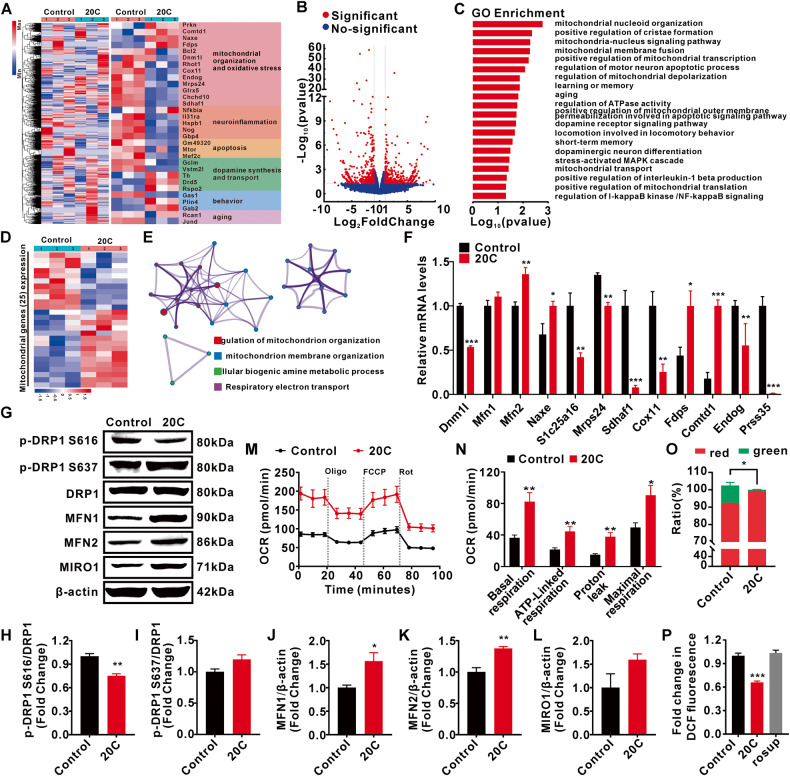
Gene expression analysis of SNc in A53T α-Syn transgenic mice treated with 20C. **A** Hierarchical clustered heatmap of gene expression profiles in SNc of A53T α-Syn transgenic mice treated by 20C or vehicle (Left). Heatmap of differentially expressed genes (DEGs) of A53T α-Syn transgenic mice treated with 20C or vehicle (control) (Right). **B** Volcano plot of DEGs between 20C and control animals. Significantly altered genes are colored in red, the rest genes are colored in blue. **C** Gene Ontology enrichment of DEGs. **D** Heatmap of differential mitochondrial genes of transgenic mice treated with 20C or vehicle (control). **E** Enrichment analysis for Gene Ontology terms among the mitochondrial genes of a gene–trait correlation module was performed using Metascape. **F** Relative mRNA levels of the mitochondrial genes in SNc. **G** Representative immunoblot of mitochondrial fusion and fission proteins in the SNc. Quantification analysis (normalized to actin or DRP1) revealed expression levels of p-DRP1 S616 (**H**), p-DRP1 S637 (**I**), MFN1 (**J**), MFN2 (**K**), and MIRO1 (**L**) in the SNc. **M** Real-time changes in oxygen consumption rate (OCR) were measured using the cellular energy metabolism analyzer (Seahorse XF24 Analyzer). Normalization of agilent seahorse XF data by in situ cell counting using a BioTek Cytation C10. **N** Quantification of basal respiration, ATP production, proton leak, and maximal respiration. **O** Mitochondrial membrane potential was stained with JC-1, and then assessed using imaging flow cytometry. **P** Intracellular ROS production was detected using DCFH-DA. (rosup was a positive control probe). Error bars are represented as SEM of mean values, *n* = 3. *p < 0.05, **p < 0.01, ***p < 0.001 *vs*. control group.

To understand the mechanism underlying the beneficial effects of 20C, we performed the genome-wide RNA sequencing on the SNc of A53T α-Syn transgenic mice treated with 20C for 40 days. 20C upregulated the genes associated with mitochondrial organization, anti-oxidative stress and dopamine synthesis. Strikingly, high levels of mitochondrial organization genes were observed in SNc of 20C-treated mice, while it inhibited the pathways related to oxidative stress and neuroinflammation (Fig. 6A). In the differentially expressed genes (DEGs), mitochondrial nucleoid organization, mitochondrial nucleus signaling pathway, mitochondrial membrane fusion and positive regulation of mitochondrial transcription are the most enriched BP induced by 20C (Fig. 6B, C). Moreover, we conducted a cluster analysis of 25 different mitochondrial genes (Fig. 6D) it was found that regulation of mitochondrial and its membrane organization are the most enriched BP induced by 20C, followed by the amine metabolic process and respiration electron transport (Fig. 6E). All these results suggested that mitochondrial organization maybe the primary BP induced by 20C.

20C led to an induction of Mfn2, Naxe and Fdps, and a decrease of Dnm1, Slc25a16, Mrps24, Sdhaf1, Cox11, Slc25a51 and Prss35, implying that 20C is involved in maintaining mitochondrial dynamic homeostasis (Fig. 6F). Hence, the key proteins involved in mitochondrial fusion and fission were then identified. Our findings show that 20C effectively increased the expression of Mfn1 and Mfn2 in SNc (Fig. 6J, K) while decreasing the expression of Drp1 phosphorylation site S616 (Fig. 6H), indicating that 20C could effectively alleviate excessive mitosis and promote mitochondrial fusion in A53T α-Syn transgenic mice, implying that mitochondrial organization maybe the primary pharmacological effects of 20C against PD. In addition, due to the sequencing analysis results revealing differential expression and regulation of the TCA cycle and respiratory chain, we further investigated mitochondrial function. We assessed the respiratory and related functions of the mitochondria by utilizing the Seahorse XF assay to measure the OCR of live cells, changes in mitochondrial membrane potential, and levels of ROS expression in cells. 20C significantly enhanced the basal respiration and maximal respiration of mitochondria, indicating an improved oxidative phosphorylation capacity of mitochondria (Fig. 6M, N). Mitochondrial membrane potential, which is essential for maintaining mitochondrial respiratory chain function, was assessed using JC-1 staining. Compared to the control group, the 20C-treated group exhibited a significant increase in the ratio of aggregated JC-1 (red fluorescence) and a decrease in the ratio of green fluorescence, indicating an elevated mitochondrial membrane potential (Fig. 6O, Fig. S10). In addition, 20C reduced intracellular ROS overproduction (Fig. 6P). These findings suggest that 20C treatment not only improves mitochondrial morphology but also enhances respiratory chain function, promoting oxidative phosphorylation and ATP generation.

### Protective effect of 20C on mitochondria in A53T α-Syn transgenic mice

**Fig. 7:**
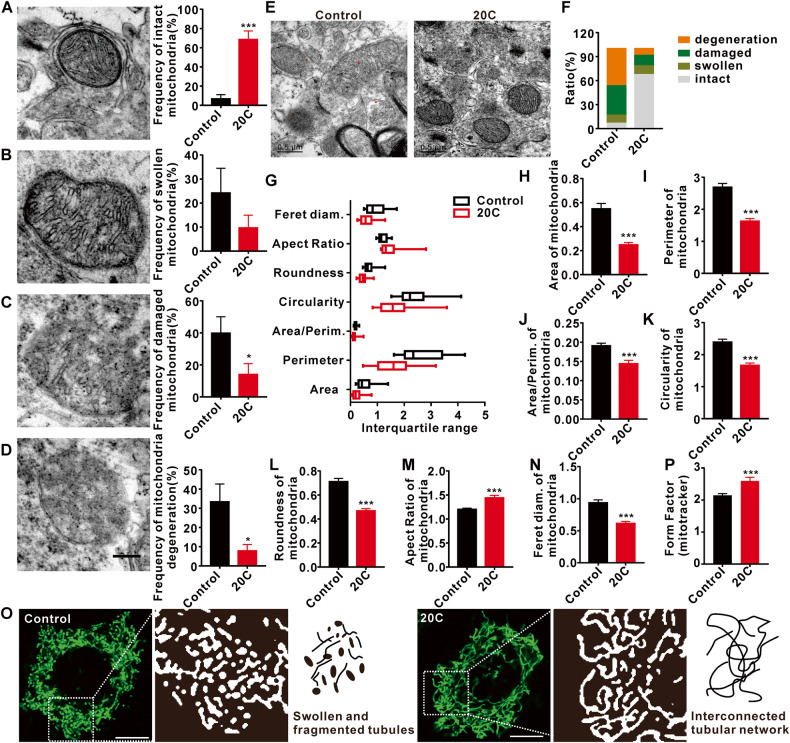
The protective effect of 20C on mitochondrial morphology. **A** Representative TEM photomicrographs (left) and percentage (right) of intact mitochondrial. **B** Representative TEM photomicrographs (left) and percentage (right) of swollen mitochondrial. **C** Representative TEM photomicrographs (left) and percentage (right) of damaged mitochondrial. **D** Representative TEM photomicrographs (left) and percentage (right) of degenerating mitochondria. Scale bar = 200 nm. **E** Ultrastructure of the mitochondria in SNc. The red spot in control represents the damaged mitochondria. Scale bar = 0.5 µm. **F** The proportion of mitochondria with different forms. **G** Box plot graph representing total mitochondrial morphological parameters. **H**–**N** Histograms for each morphological parameter of mitochondria: area, perimeter, area/perimeter ratio, circularity, roundness, aspect ratio, and Feret diameter. **O** Photomicrographs in green demonstrate changes in mitochondrial morphologies in control and 20C group; Photomicrographs in white are the enlarged images of the designated square boxes in the green photomicrographs. Mitochondria in 20C existed as an interconnected tubular network, whereas mitochondria in control appeared as swollen tubes and fragmentation (short separate tubes & swollen tubes). **P** Histograms for each morphological form factor presented in (**O**). (Error bars are represented as SEM of mean values, *n* = 3. *p < 0.05, ***p < 0.001 *vs*. the control group).

In light of the regulation of mitochondrial organization by 20C, their ultrastructure in SNc was revealed by TEM. As shown in Fig. 7, mitochondria were divided into four states according to their structure: intact, swollen, damaged, and degenerated mitochondria. The statistical analysis showed that compared with the control group, the percent of intact mitochondria in the 20C groups was significantly increased (Fig. 7A), while the percent of damaged mitochondria (Fig. 7C) and degenerated mitochondria (Fig. 7D) were significantly reduced. Swollen mitochondria in the 20C showed the downward trend, but failed to reach statistical difference in this study (Fig. 7B).

Figure 7E is a representative mitochondrial ultrastructure of the control group, which are swollen and oversized, with matrix and cristae loss and leakage, and some mitochondria that have degenerated to no visible outline. While the mitochondria of the 20C group maintained their normal shape and the outline was clearly visible. Their statistical results of the classification of the two groups of mitochondria, as shown in Fig. 7F, showed that the contents of intact, swollen, damaged, and degraded mitochondria in the control group accounted for 6.67%, 10%, 36.67%, and 46.67%, respectively. In 20C group, the intact mitochondria accounted for 67.39%, the swollen mitochondria accounted for 10.86%, the damaged mitochondria accounted for 13.04%, and the degenerated mitochondria accounted for 8.69%. We observed an increase in the length-to-width ratio and a decrease in the roundness of mitochondria in the 20C group (Fig. 7G), indicating that 20C could effectively improve the morphology of mitochondria and maintain their normal structure. Separate analysis of the indexes describing the morphology and structure of mitochondria showed that the area, perimeter and ratio of the group treat with vehicle (later referred to as the control group) were significantly higher than those of the 20C group (Fig. 7H–J), the roundness analysis showed that the control group was significantly larger than the 20C group (Fig. 7K, L), and the aspect ratio of the control group was significantly lower than that of the 20C group (Fig. 7M). At the same time, diameter analysis also showed that the control group was significantly larger than the 20C group (Fig. 7N). And then, we analyzed the observations related to fusion and fission of mitochondrial morphology changes using mito-tracker staining (Fig. 7O, P). The results demonstrated that the control group without treatment exhibited significant cellular fragmentation, whereas treatment with 20C restored mitochondrial morphology and maintained a continuous elongated tubular shape. These findings suggest that 20C effectively delayed mitochondrial damage and degeneration in A53T transgenic mice while maintaining mitochondrial morphology and function.

## Discussion

α-Syn aggregation is a critical pathophysiological factor in the progression of PD, which was identified as the most abundant protein in Lewy bodies and play an important role in many cellular processes [34]. Therefore, targeting α-Syn aggregation is expected to alleviate the progression of PD [35].

As a promising molecule screened from the *Gastrodia elata*, 20C has been found to improve PD-like motor dysfunction caused by chemical agents and decrease the levels of pathological α-Syn in different forms of brain [18, 25, 36, 37]. However, whether these improvements are result from the interaction between 20C and α-Syn directly is still obscure. A53T mutation promotes the formation of α-Syn fibril and enhances the proliferation and toxicity of fibril [38] in vitro. Here, TEM analysis confirmed that 20C could inhibit the aggregation of A53T α-Syn. It was found that more than 50% A53T α-Syn mutant amyloid protein is inhibited from the ratio of 0.7:1 (A53T α-Syn: 20C). The inhibitory activity of 20C showed the concentration-dependent manner even at ratio of 7:1 (A53T α-Syn: 20C).

The results of computer-aided molecular docking and kinetic simulation show that 20C may bind to the β-sheet toxic region of α-Syn fibril structure. This direct interaction is confirmed by circular dichroism experiments. Application of 20C changes the secondary structure conformation of α-Syn, resulting in the decrease of β-sheet and the increase of α-helix in the original α-Syn conformation. This is very important because the β-sheet structure is the main toxicity secondary structure of α-Syn, which is easy to form amyloid protein. In the inherently disordered protein α-Syn, the hydrophobic and electrostatic interactions produced by protein residues prevent the contact between proteins, thus maintaining the physiological state and performing physiological functions [39]. The failure of α-Syn misfolding and aggregation causes the protein to turn to the pathological state. The hydrogen bonding and hydrophobic interaction between 20C and α-Syn β-sheet residues maintain the physiological state of α-Syn, which may be the fundamental reason why 20C inhibits the formation rate and quantity of α-Syn fibril. The MST experiments analyzed from the biophysical level show that there is a direct interaction between 20C and α-Syn fibril, and it is a strong affinity, stable binding and strong competitive advantage.

20C displayed low toxicity for neural cells and good cellular permeability. Indeed, 20C treatment with a concentration of 10 μM significantly reduced the number of α-Syn inclusions. These data prompted us to evaluate the efficacy of 20C in the treatment of α-Syn toxic injury in vivo. First, we selected a well-established animal model of Parkinson’s disease, the PD-like pathological model of A53T α-Syn mutation. After oral administration of 50 mg/kg for 40 days, we observed a significant decrease level of α-Syn variants (aggregates and nitrified α-syn) in SNc and Str of mice, and a significant improvement in motor behavior disorder. However, the ultimate aim of anti-PD therapy is not to interfere with α-Syn aggregation itself, but to prevent neurological degeneration associated with α-Syn. As described in the recent literature, α-Syn levels may affect the vertical decline of the network of exercise-related functional connections. Then, we analyzed the overall brain functional connection, 20C enhanced brain FC are located in the SNc and cortex-striatum connection network, mesencephalic marginal-striatum ring connection network and extended brain stem (midbrain-pons-thalamus). Furthermore, the correlation analysis between ALFF and behavioral performance validated that enhanced ALFF in the SNc was significantly positively correlated with shortened climbing time, shortened time to cross the balance beam, and increased grip strength in A53T transgenic mice. The connectivity results of brain regions also demonstrated that 20C enhanced the connectivity between SNc and other brain regions. These brain regions are closely related to the regulation of motor function, and although more mechanisms may be involved, our findings support the view that 20C enhances the brain functional connection between SNc and motor-related brain areas by inhibiting α-Syn aggregation.

In addition, 20C protected against the loss of dopaminergic neurons, decreased the number of Lewy bodies, and improved the shrinkage and degeneration of neuronal nuclei. In fact, in the early stage of neuronal degeneration, mitochondrial dysfunction is prior to structural changes and the loss of dopaminergic neurons before motor behavior disorder. The structural damage of mitochondria caused by abnormal α-Syn deposition is involved in PD mitochondrial pathology. 20C protected the morphological damage of mitochondria, kept the bilayer membrane intact, the matrix was uniform, the cristae was arranged neatly, the ratio of length to width increased and the roundness decreased. Intact mitochondrial morphology is characterized by increased activity, which may support continuous neural transmission, and the morphological changes of mitochondria are closely related to functional compensation.

Oxidative stress, mitochondrial fission and fusion, as well as motor behavior and memory are the main biological processes of regulatory disorders in PD. When α-Syn is overexpressed or mutated, the homeostasis in neurons changes quickly. The first change is the mitochondria involved in bioenergy regulation. Mutated or overexpressed α-Syn fibrils or oligomers are anchored to the cell membrane of mitochondria. By destroying and splitting the structure of mitochondria or changing the permeability of mitochondrial membrane to increase the level of reactive oxygen species in mitochondria, hence, disrupt mitochondrial function and inhibit mitochondrial dynamics, which is characterized by increased mitochondrial fission, decreased energy production and decreased expression of marker proteins. With the persistent imbalance of mitochondrial biological function, the degradation of α-Syn is blocked, and the continuous increase of reactive oxygen species will drive the toxic α-Syn conformation, thus forming a vicious circle. In this study, the changes of mitochondrial genes observed at the transcriptional level supported the protective effect of 20C on mitochondrial dysfunction. 20C regulates the expression of genes Dnm1l, Mfn1, Mfn2, and Mrps24 [40] related to mitochondrial division and integrity, suggesting that 20C may maintain the homeostatic balance between mitochondrial fusion and fission dynamics. α-Syn plays a central role in regulating the dynamics and function of mitochondria [41]. The up-regulation of α-Syn level may promote fission through the Drp1-dependent pathway and subsequent changes in mitochondrial morphology, resulting in reduced fusion and eventually interference in the mitochondrial fission-fusion cycle [42]. The results of the expression of key proteins to maintain the homeostasis of mitochondrial dynamics, improving mitochondrial respiration function, preserving mitochondrial membrane potential, and inhibiting excessive ROS production confirmed our conjecture that 20C effectively alleviate the excessive division of mitochondria and promote the fusion of mitochondria in α-Syn A53T transgenic mice, thus improving the dynamics of mitochondria and maintaining the morphology and function of mitochondria. The differential expression of migraine susceptibility gene Naxe [43], mitochondrial transporter encoding genes Slc25a16 [44] and Slc25a51 [45], mitochondrial respiratory function related gene Sdnaf1 [46] and Cox11 [47], regulation of cholesterol biosynthesis gene Fdps [48], methyltransferase activity gene Comtd1 [49], regulation of apoptosis gene Endog [50], and Prss35 [51], a gene highly expressed in renal fibrosis, may be the compensatory increase after mitochondrial injury. But the effect of 20C on mitochondrial damage compensation is still not clear, which needs to be further explored.

**Fig. 8:**
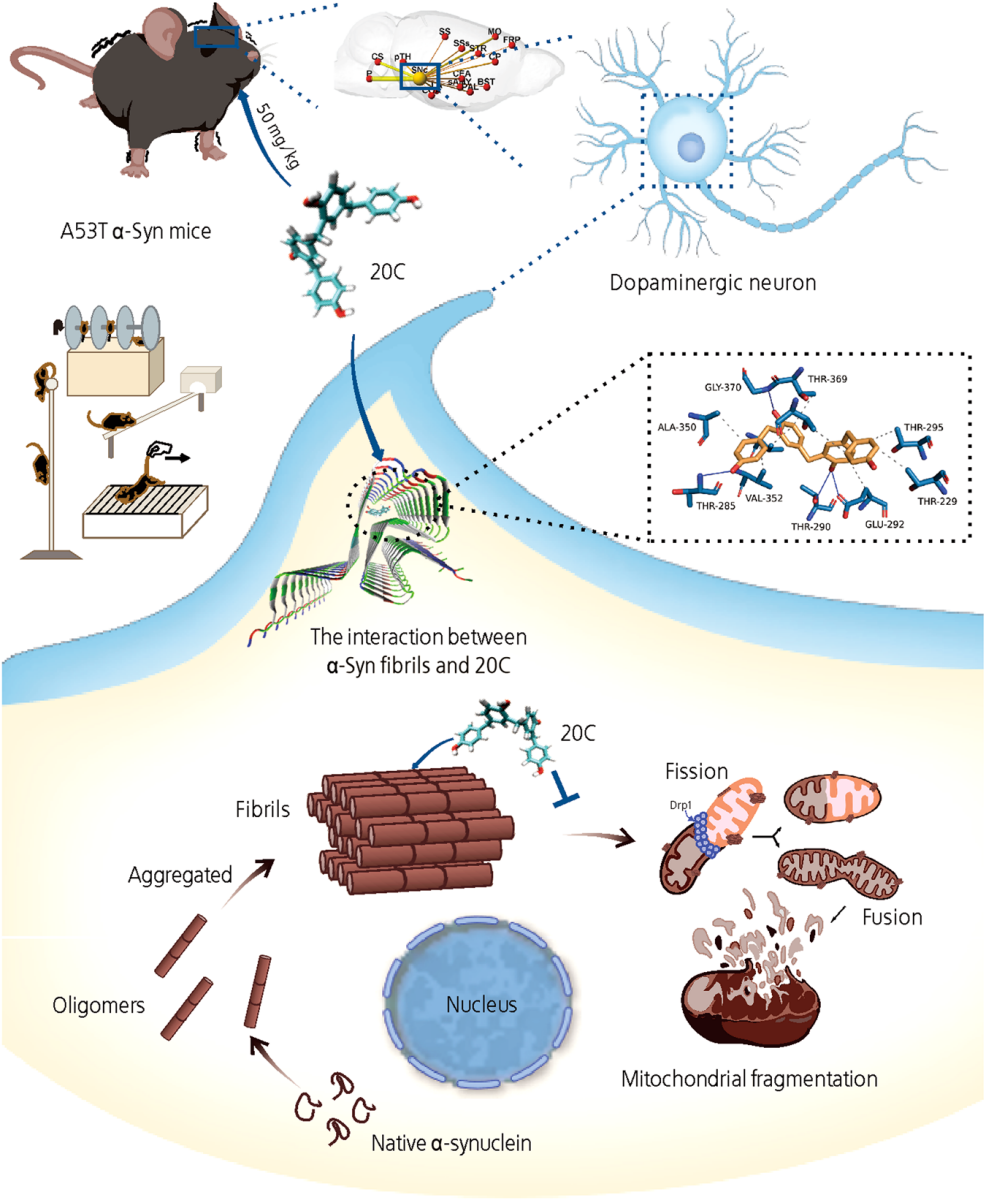
20C ameliorates mitochondrial dysfunction and PD process by inhibiting α-Syn aggregation. 20C binds to the core toxic β-sheet structure of α-Syn fibrils, decrease the content of β-sheet structure of α-Syn, thus inhibit the accumulation of α-Syn, further inhibit the maturation of α-Syn, improve the dysfunction of mitochondria, maintain the homeostasis of mitochondrial dynamics, protect dopamine neurons, and play a biological role in the treatment of PD.

In conclusion, 20C ameliorates mitochondrial dysfunction and alleviates PD process by inhibiting α-Syn aggregation (Fig. 8). 20C binds to the core toxic β-sheet structure of α-Syn fibrils, decrease the content of β-sheet structure of α-Syn, thus inhibit the accumulation of α-Syn, further inhibit the maturation of α-Syn, improve the dysfunction of mitochondria, maintain the homeostasis of mitochondrial dynamics, protect dopamine neurons, and play a biological role in the treatment of PD.

## Methods

### Materials

The sources of antibodies, chemicals, assay kits, and instrument used in this study are listed in Supplementary Table S2.

### Cell culture

PC12 cells were cultured in RPMI 1640 medium supplemented with 10% horse serum and 5% fetal bovine serum (FBS). SH-SY5Y cells and H4 cells were cultured in Dulbecco’s modified eagle medium (DMEM) supplemented with 10% FBS. These cells were cultured at 37 °C in 5% CO_2_ incubator and passaged every two days.

### Determination of anti-PD activities of 20C in vitro

A total of three cellular models were introduced to observe the anti-PD activities of 20C, including extracellular α-Syn aggregates (40 μM) model, chemical agent rotenone (4.0 mM) and serum free induced cellular model. The concentration 20C was set from 2.5 μM to 40 μM. The cell viability was quantified by MTT after 24 h of treatment. The cell viability of control group was set as 100%.

### Transmission electron microscopy (TEM)

The samples of A53T α-Syn protein (70 μM) with or without of 20C (100 μM) at different time points were immediately placed on the carbon-coated copper grid, and then the sample was dyed with 2% (w/v) uranium acetate solution. The images were obtained under 120 kV acceleration voltage by TEM. A total of 25 images was collected from each sample to obtain representative images.

### Thioflavin-T (Th-T) determination

Each well of a 96-well sealed plate containing 70 μM A53T α-Syn, 40 μM Th-T and 2 mm diameter polytetrafluoroethylene beads with or without of 20C from 1 μM to 200 μM in a total volume of 100 μL. The plates were mixed and incubated on a THZ-D thermostatic oscillator at 100 rpm and 37°C. The fluorescence value of Th-T was measured every 2 h in the first 12 h and once a day until the fourth day, excited by 430–450 nm filter, and the transmitted signal was collected by 480–510 nm filter using a EnSpire Multimode Reader. To observe the effect of 20C on different stages of aggregation, we added 20C (100 μM) at 4 h, 8 h, 12 h, 24 h, 36 h in the above protein-Th-T system and measured the fluorescence after 96 h incubation.

### Nanometer particle size analysis

The end products of A53T α-Syn aggregation or the products after proteinase K digestion were collected and diluted to the final volume of 1 mL with ultra-pure water. Zetasizer Nano ZS was used to measure the sample and record the light scattering of all particles in this solution. All samples were measured repeatedly for eight times, and the number of particles with different sizes in the system were simulated and calculated by Advanced Protein Feature module.

### Circular dichroism (CD)

The end products of A53T α-Syn aggregation were diluted to 0.1 mg/mL (7.14 μM) with ultra-pure water, and the concentration of 20C was 71.4 μM. The scanning wavelength is 200–260 nm with the scanning speed is 50 nm/min, and the response time is 1 s. The near ultraviolet (200–245 nm) CD signal reflecting the secondary structure of protein polypeptide chain was selected for mapping.

### Microscale thermophoresis (MST)

The A53T α-Syn fibrils were labeled with RED-NHS. After buffer replacement, protein purification and balance, giving a 0.1428 μM solution of labeled protein. All measurements were conducted using Monolith NT.115 instrument at 25 °C. Assays were conducted at 60% IR-laser power and MST powers. Compounds 20C (32.768 mM) and α-Syn antibody (8 mM) were initially dissolved in PBS containing 8% DMSO to afford stock solutions. During the MST experiments, these compounds were serially diluted 1:1 with buffer containing 8% DMSO to ensure a constant DMSO concentration throughout the dilution series. Fnorm values from analysis of the T-jump (8.28 s–9.08 s) were used for fitting.

### Cell death measurement

To investigate the impact of proteins incubated in vitro on the cytotoxicity of SH-SY5Y cells, we exposed these cells to extracellularly cultured A53T α-Syn protein (at a final concentration of 5 μg/mL) or proteinase K-digested protein for a duration of four days, while also considering the presence of 20C. This treatment was continued for a total of 7 days. Subsequently, the cellular viability was quantitatively assessed using MTT reagent, with the untreated group serving as the control at 100% viability.

### Characterization of 20C-fibril interaction

#### α-Syn fibrils model

In order to predict the binding model of 20C to A53T α-Syn fibrils and calculate the binding free energy, we used the PDB structure 2N0A. We adopted a simplified model of α-Syn fibril, which contains only 34–99 residues of 2N0A instead of the full-length α-Syn chain, to increase the probability of 20C entering the structural center. The simplified model has passed the molecular dynamics simulation test and has been used as an effective model to replace the full-length α-Syn fibril in all calculations [24]. The β-synuclein was built using the SwissModel server, and a protein structure simulation was performed. The selected protein structure for the β-synuclein showed homology of 68.217%, compared to the crystal structure of the α-Syn protein (PDB ID: 2N0A).

#### Prediction of 20C-fibril interaction binding sites

In order to predict the interaction between 20C and α-Syn fibrils, we used Discovery Studio (DS) 2016 software to simulate molecular docking. The structure of 20C is constructed by small molecules module. The simplified model of fibril is prepared by Macromolecules module. The docking site selection is prepared by the Receptor-Ligand Interactions module to generate a sphere with a radius of 10 Å in the fibril structure. LibDock (hot spot matching) is used in the Receptor-Ligand Interactions module for docking program.

#### 20C-fibril molecular dynamics simulation

A total of three 20C-fibril complexes were simulated by all-atomic molecular dynamics using AMBER18 software. Before the simulation, the charge of the small molecule was calculated by the antechamber module and the Hartree-Fock (HF) SCF/6-31G^*^ of the gaussian 09 software [52, 53]. After that, the small molecule and protein were described by GAFF2 small molecular force field and ff14SB protein force field [54], respectively. Each system uses LEaP module to add hydrogen atoms to the system, adds a truncated octahedral TIP3P solvent box at the distance of 10 Å [55], and adds Na^+^/Cl^-^ to the system to balance the charge of the system, and finally outputs the topology and parameter files for simulation.

### Binding energy calculation

The binding free energies between proteins and ligands in all systems were calculated by MM/GBSA method [56, 57]. In this study, the MD trajectory of 45–50 ns is used as the calculation, and the specific formula is as follows:

1 $$\begin{array}{l}{\Delta {\rm{G}}}_{{{\rm{bind}}}}={\Delta {\rm{G}}}_{{\rm{complex}}}-\left({\Delta {\rm{G}}}_{{\rm{receptor}}}+{\Delta {\rm{G}}}_{{\rm{ligand}}}\right)\\\qquad\qquad=\,{\Delta {\rm{E}}}_{{\rm{internal}}}+{\Delta {\rm{E}}}_{{\rm{VDW}}}+{\Delta {\rm{E}}}_{{\rm{elec}}}+{\Delta {\rm{G}}}_{{\rm{GB}}}+{\Delta {\rm{G}}}_{{\rm{SA}}}\end{array}$$

In formula (1), ΔE_internal_ represents internal energy, ΔE_VDW_ represents van der waals interaction and ΔE_elec_ represents electrostatic interaction. The internal energy includes bond energy (Ebond), angular energy (Eangle), and torsion energy (Etorsion), and ΔG_GB_ and ΔG_GA_ are collectively called solvation free energy. Among them, GGB is polar solvation free energy and GSA is non-polar solvation free energy. For ΔG_GB_, the GB model developed by Nguyen and other researchers is used to calculate (igb = 2). The non-polar solvation free energy (ΔG_SA_) is calculated based on the product of surface tension (γ) and solvent accessibility surface area (surface area, SA), “GSA = 0.0072 × solvent SASA [58]. Entropy change is ignored in this study because of high consumption of computing resources and low precision [56, 59].

### Animals

All animal care and experimental procedures complied with the principles outlined in the NIH Guide for the Care and Use of Laboratory Animals and were approved by the Institutional Animal Care and Use Committee of the Peking Union Medical College and Chinese Academy of Medical Sciences (Ethics number: 00003907).

Human A53T α-Syn over-expressing [B6;C3-Tg(Prnp-SNCA*-A53T)83Vle/JNju] mice [60, 61] and wild-type mice of the same age were purchased from the Model Animal Research Center of Nanjing University and raised in SPF Biotechnology Co., Ltd. All animal were maintained in cages (five animals per cage) under constant temperature and humidity and exposed to a 12:12 h light–dark cycle with unrestricted access to tap water and food.

A53T α-Syn transgenic mice (male, aged 12 months, weight = 31 ± 2 g, *n* = 24) were randomly divided into two groups: Vehicle and 20C (*n* = 12 per group). In our previous study, the optimal dose of 20C in the treatment of PD was found at 50 mg/kg in the MPTP/p mouse model [25], which was also used in this study.

### Pole test

The limb coordination and adhesion ability of the mice in the pole test were evaluated by rod climbing time. The device consists of a piece of wood with a long 50 cm with a diameter of 1 cm, wrapped around gauze to prevent animals from slipping, and the bottom was placed in a feeding cage and covered with padding to prevent mice from being injured. Record the time that the mice crawled from the top of the stick to the bottom. Measure three times in succession, each time at intervals of 30 min. The average value of the three measurements was selected for data analysis.

### Beam walking test

The limb coordination and adhesion ability of mice in the beam walking test were evaluated by the time it passed through the beam. The device consists of 80*1.5*1 cm poles, with a width of 1.5 cm and a height of 40 cm. The mice’s dark nature was used to induce it to move from one end of the beam to the other by placing a cassette at one end of the pole. Placed the mice at one end of the beam and time when it consciously climbed over the beam. Measure three times in succession, each time at intervals of 30 min. For data analysis, the average of the three measurements was chosen.

### Rotating rod test

The rotating rod test was a behavioral experiment used to evaluate the neuromotor function of rodents. In this experiment, the experimental mice were placed on a roller with a diameter of 30 mm and began to rotate, and gradually increased from 1 rpm to 30 rpm within 300 s. Each mouse was measured three times with an interval of at least 30 min, and the average value was recorded and sorted out.

### Grip strength test

Grip testing equipment was used to measure grip strength. The animal was positioned in front of a metal net. Drag the mice from one end of the device to the other using the same amount of external force, and measure the force required to pull the mouse away, revealing the mice’s forelimb grip. As the correct measurement result, the arithmetic mean of the three measured values is determined.

### Open-field test

Open-field test was used to evaluate the autonomous activity of animals in unfamiliar environments. The test was conducted in a 50*50*40 cm box surrounded by black coating, with 16 squares at the white bottom. The mice were placed in the central area and allowed to explore freely for 5 min. Smart 3.0 software recorded its activity trajectory and the total exercise distance.

### Novelty suppressed feeding test (NSFT)

NSFT was used to evaluate the anxiety and feeding desire of animals in unfamiliar environment after fasting. Place the mice outward from a corner of the device and record the period during which the mice begin to feed within 8 min. Food intake was measured by the mice biting the food rather than fiddling with or sniffing it.

### Sucrose preference test (SPT)

SPT reflects the lack of pleasure by measuring the percentage of sucrose water consumed by animals in a certain period. Before the experiment, all mice were given 1% sucrose water and normal drinking water for 2 days, and the water was banned for 12 h. The experiment lasted 6 h, in which 1% sucrose water and the position of the normal drinking water bottle were exchanged after 3 h. Weigh and record two kinds of water bottles before and after the test. 1% sucrose water consumption percentage (%) = 1% sugar water/(1% sugar water + normal drinking water) × 100%.

### Cell infection

PRLenti-EF1a-mcherry-P2A-Puro-CMV-SNCA (A53T)-HA lentivirus was used to infect SH-SY5Y cells. After two weeks of screening by puromycin, these cells (SNCA (A53T)-SY5Y cells) were used in the experiment. pAAV-hSyn-SNCA(A53T)-WPRE lentivirus was used to infect SH-SY5Y cells for determining changes in mitochondrial membrane potential. The transfection efficiency was validated using qPCR method (Fig. S9A).

H4 cells were transiently infected with pRLenti-EF1-Puro-CMV-SNCA (A53T)-EGFP-3xFLAG-WPRE. After infection, the cells were divided into vehicle group (control) and 20C group (given 10 μM 20C). After 24 h of incubation, the cell suspension was collected, the positive cells (A53T-EGFP) and α-Syn inclusions patterns were screened and counted by imaging flow cytometry.

### Experimental cell treatment

MG132 (10 μM) and 3-Methyladenine (3-MA) (5 mM) were administered to SNCA(A53T)-SY5Y cells and incubated for 24 h to examine the role of protein degradation on the levels of α-Syn induced by 20C.

### Thioflavin S staining

SNCA (A53T)-SY5Y cells were plated on 24-well plate-sized cover glass. After 24 h, the cells were divided into vehicle group (control) and 20C group (10 μM). The second day, they were fixed with 4% paraformaldehyde for 20 min. After PBS washing for 3 times, the cells were stained with 0.5% Th-S dye solution (dissolved in 50% ethanol) for 5 min, 70% ethanol hydration for three times until the dye solution color faded. The image was collected by confocal microscope, and the Th-S positive staining was considered as α-Syn aggregates. According to the number of intracellular aggregates, the positive cells were divided into 0, 1, 2, 3, 4 and more than 5 aggregates and scored. At least 90–100 cells were counted in each group.

### Imaging flow cytometry

The formation of α-Syn inclusions was analyzed using an imaging fow cytometer. The cells were washed and finally re-suspended in 50 µL cold FACS bufer (PBS with 2% FBS). One group served as a negative control to exclude autofluorescence, the other one was used for determining the effect of 20C on the levels of α-Syn inclusions. To generate a compensation matrix, the EGFP-positive sample was additionally measured using all emission channels. The software IDEAS® (Merck Millipore, Billerica, USA) was used for data analysis. A total of three independent experiments for each condition were performed.

### Immunohistochemical staining

Mice were perfused with 4% paraformaldehyde. After the brain was removed, it was fixed for 2 days, then transferred to isopentane for cryopreservation and frozen in liquid nitrogen for 15 s. A series of coronal sections were collected with a thickness of 20 μm. The coronal sections containing the pars compacta of substantia nigra were immunohistochemical to evaluate the expression of tyrosine hydroxylase (TH). The slices were kept slightly boiling for 10 min in citrate antigen repair solution, treated with 0.5% Triton-X-100 and inactivated by 3% hydrogen peroxide, sealed with 5% bovine serum albumin (BSA) and incubated with target protein antibodies overnight in a wet box at 4 °C. The next day, incubated with the second antibody (1:500) at room temperature for 2 h. The sections were stained with DAB and the images were collected by microscope. For Lewy body staining, digestion with proteinase K was followed by immunohistochemical follow-up. The cell count for the substantia nigra was obtained using a dot counting method to determine the number of positive neurons.

### Immunofluorescence

Immunofluorescence was performed on the coronal brain sections containing the striatum and the pars compacta of substantia nigra, respectively, which was the same as the above experimental steps except for the inactivation. The next day, incubated with immunofluorescence second antibody and Hochest33342 in the dark at room temperature for 2 h. The fluorescence was observed and captured by confocal microscope. The cell count for the substantia nigra was obtained using a dot counting method to determine the number of positive neurons, while the mean optical density value of TH-positive staining in the striatum, α-Syn intensity, and 5G4 intensity were quantified using Fuji software.

### Nissl staining

After fixation by 4% paraformaldehyde, brains were embedded in paraffin following standard procedures and serially sectioned at 4 μm for staining. Next, the slices were immersed in 2% thionine for 3 min, ultrapure water for 10 s (twice), 100% ethanol for 10 s, 95% ethanol for 10 s, 85% ethanol for 10 s, 70% ethanol for 10 s, xylene for 2 min, and 100% ethanol for 10 s and then washed with ultrapure water for 5 min. Finally, the slices were sealed with neutral gum after desiccation.

### Western blot

Coronal sections ranging from 3 to 4 mm from the vertical plane of the bregma point were selected and the substantia nigra region was dissected for protein extraction. Tissue or cells were cleaved in a cold cleavage solution containing 50 mM Tris-HCl (pH 7.4), 150 mM NaCl, 1 mM EDTA, 1% NP-40 (RIPA) and 1% protease inhibitor and 1% phosphatase inhibitor. The protein concentration was determined by BCA method. Cell culture medium was collected and centrifuged at 4 °C at 10,000 × *g* for 20 min to remove dead cells and debris and the supernatant concentrated using 3 kD centrifugal filter units (Millipore) for Western blot. 10% SDS-PAGE was selected for gel electrophoresis, and the protein was transferred to 0.45 μm PVDF membrane by wet electroporation. Blocked with 5% BSA and hybridized with target protein antibody overnight at 4 °C. Wash the membrane in TBS containing 0.1% Tween-20 buffer (TBST) for 10 min each time, then hybridize the corresponding secondary antibody at room temperature, wash the membrane 3 times in TBST for 10 min each time. The protein bands were visually captured by Image Quant LAS 4000, and the comprehensive density was analyzed by Image J.

### Mitochondrial morphological analysis

The ultrastructure of mitochondria was evaluated using Image J, and parameters including density, area, perimeter, and area/perimeter ratio were quatified. 20 pictures of each group were analyzed, and their morphological measurement included the following parameters: aspect ratio (AR), computed as [(major axis)/(minor axis)], which reflects the length-to-width ratio; circularity [4π·(surface area/perimeter)] and roundness [4·(surface area)/(π·major axis)], which are two-dimensional indexes of sphericity with values of 1 indicating perfect spheroids; and Feret diameter, which represents the longest distance (µm) of a selected mitochondrion. The ultrastructural defects of mitochondria were quantified according to their appearance, which were divided into the following categories: intact mitochondria, normal appearance of crest; swelling of mitochondria, integrity of double membrane, swelling of crest; damage of mitochondria, membrane damage, swelling and shedding of crest or irregular morphological damage; and degenerated mitochondria, the boundary of double membrane disappeared, the matrix was exposed and the crest disappeared [62].

### Mito-tracker staining

SNCA (A53T)-SY5Y cells were plated on 24-well plate. After 24 h, the cells were divided into vehicle group (control) and 20C group (10 μM). The second day, mito-tracker staining was performed. The cells were incubated at 37 °C while avoiding light with 100 nM mito-tracker medium for 30 min. The staining solution was removed, and the cells were washed three times with medium. Subsequently, the cells were fixed with 4% paraformaldehyde for 10 min, washed three times with PBS, and treated with 0.2% Triton-X-100 for 5 min. The cells were then washed three times with PBS and blocked with 3% BSA at room temperature for 30 min. The mitochondrial morphology was analyzed using Image J with a 2D mitochondria analyzer plugin.

### Quantitative real-time PCR

Total RNA was extracted from SNc using TRIzol reagent. Quantification and integrity analysis of total RNA was performed by running 1 µl of each sample on NanoPhotometer N50 Touch. The cDNA was prepared by reverse transcription. The relative expression of mRNAs was determined by the SYBR Green PCR system. The relative expression of genes of interest was calculated by comparative Ct method and β-actin was used as an endogenous control. β-actin RNA was chosen as the housekeeping gene. Sequences of the primers used for real-time qPCR in this study were as Supplementary Table S3.

### Whole genome RNA sequencing analysis

Total RNA was extracted from SNc with TRIzol reagent. The total RNA quality was tested by Nanodrop 2000, and then sequenced on Novaseq 6000 PE150 platform. The off-machine data (Raw Data) of high-throughput sequencing was filtered to get high-quality data (Clean Data). Using HISAT2 to compare the similarity between clean data and reference genome (mapping), the location information of reads on the reference genome and the characteristic information of sequencing samples are obtained, and the bam file is generated. Based on the count number of genes, DESeq2 was used to analyze the differentially expressed genes (DEG) at the gene level. The criteria for defining differential expressions are: simultaneously satisfy: |log2 (fold change)| > 1 and *p* < 0. 05. Gene ontology (GO) and pathway annotation enrichment analysis are based on NCBI COG (https://www.ncbi.nlm.nih.gov/ COG/), gene ontology database (http://www.geneontology.org/) and KEGG pathway database (http://www.genome.jp/kegg/). The gene expression patterns were analyzed by hierarchical cluster analysis with Cluster software and Java Treeview software.

### Measurement of cellular metabolism

Cellular oxygen consumption rate (OCR) was measured using a Seahorse XF24 analyzer. The XF24 sensor cartridge was activated with 1 ml of XF calibrant solution per well for 12 h at 37 °C. SNCA (A53T)-SY5Y cells (1 × 10^4^ cells/ml) were seeded onto XF24 cell culture microplates and treated with vehicle group (control) or 20C group (10 μM) for 24 h. One hour before measurement, the culture medium was changed to serum-free and bicarbonate-free DMEM supplemented with 10 mM glucose, 5 mM pyruvate, and 5 mM glutamine. After incubation for 1 h at 37 °C in a non-CO_2_ incubator, steady-state and post-intervention analyses were performed. Respiration was assessed by injection of oligomycin (1.5 µM) to inhibit the mitochondrial ATP synthase, carbonyl cyanide-p-trifluoromethoxy-phenylhydrazone (FCCP; 2 µM) to collapse the mitochondrial membrane potential, and rotenone (0.5 µM) to inhibit the respiratory chain. The OCR was normalized to total cell amount used by Cytation C10.

### Reactive oxygen species assay

SNCA (A53T)-SY5Y cells were plated on 96-well plate. After 24 h, the cells were divided into vehicle group (control) and 20C group (10 μM). The second day, cellular reactive oxygen species were detected and quantified using a reactive oxygen species (ROS) assay kit, following the manufacturer’s instructions.

### Mitochondrial membrane potential assay

SNCA (A53T)-SY5Y cells were plated on 96-well plate. After 24 h, the cells were divided into vehicle group (control) and 20C group (10 μM). The second day, mitochondrial membrane potential were detected and quantified using a mitochondrial membrane potential assay kit with JC-1, following the manufacturer’s instructions. Results were performed by imaging flow cytometry analysis.

### MRI data acquisition and processing

MRI datasets were generated on PharmaScan 70/16 (Bruker BioSpin, Ettlingen, Germany), and the image acquisition is collected by ParaVision_6.0.1 software (Bruker BioSpin GmbH). Animals were initially anesthetized continuously with 2% isoflurane in a mixture of 70/30% N_2_/O_2_ continuous gas to stable positioning. The head is fixed in a dedicated animal cradle, including tooth and ear strips, to reduce motion artifacts throughout the scan; then isoflurane is reduced to 1.5% at the beginning of the scan. At the beginning of each MRI examination, a FieldMap and continuous local gaskets are used to improve the uniformity of the magnetic field, and then T2-weighted TurboRARE sequences are used as anatomical reference scans, functional MRI was then acquired with a free induction decay echo-planar imaging (FID-EPI) sequence with 300 repetitions with a field of view (FOV) of 20 × 20 mm^2^, 40 sections with a thickness of 0.35 mm at intervals of 0.05 mm, matrix dimension of 64 × 64, repetition time (TR) = 2000 ms, echo time (TE) = 15 ms.

The mouse brain atlas informed for functional data. We selected the followed brain regions: frontal pole, cerebral cortex (FRP), somatomotor areas (MO), somatosensory areas (SS), supplemental somatosensory area (SSs), striatum (Str), caudate putamen (CP), globus pallidus (PAL), bed nuclei of the stria terminalis (BST), striatum-like amygdalar nuclei (sAMY), cortical amygdalar area (COA), lateral amygdalar nucleus (LA), central amygdalar nucleus (CEA), part of thalamus (pTH), pons (P) and superior central nucleus raphe (CS) (midbrain limbic, extended brain stem) connected with the pars compacta nigra (SNc) for the independent analysis. In addition, we selected the inferior receptacle Postsubiculum (POST) [32], Parasubiculum (PAR) [33], COA [63], periventricular thalamic nucleus (PVp) [28], endopiriform nucleus (EP) [26], medial preoptic nucleus (MPN) [29], parabrachial nucleus (PB) [30] and koelliker-fuse subnucleus (KF) [31] were used to analyze the interconnection and independence of the nodes in the brain region of interest (ROI). These areas are closely related to motor and non-motor concomitant symptoms of PD, such as cognitive impairment, sleep disorders, depression, anxiety, pain, among others.

To examine the relationship between the assessed motor behaviors and the brain changes induced by 20C, we performed Pearson (data fit normal distribution) or Spearman (data not fit normal distribution) correlation analyses of the behavioral test index and ALLF/ReHo values of the brain regions that showed significant differences. *p* < 0.05 was considered significant.

Brain functional data set extraction, using dcm2nii (2MAY2016) to convert the original data into nifti format files, voxel magnification 10 times, taking the middle layer as the reference layer for time layer correction, realign: the temporal processed volumes of each subject was re-aligned to the mean volume to remove any head motion, and a mean image was created over the 300 re-aligned volumes, after cephalometric correction, all the subjects’ data was registered to the standard space, and the ants (1.9.2) toolkit was used for registration according to the two-step registration method. Register the function image to Allen Mouse Brain [64] through T2 and average function image. Then, we use SPM12 and restplus V1.2.8-130615 toolkits to perform Gaussian smoothing (smoothing kernel = 6 mm) and filtering (range 0.01–0.08 Hz) on the registered data. Finally, after regression covariable analysis and de-linearization trend, we get the data to be used for analysis.

In order to evaluate the functional connection of the whole brain region, the whole brain was divided into 151 seed points, the correlation coefficients among the seed points were calculated, and zFC was obtained by Fisher-z standardization. MATLAB R2013b (The MathWorks, Inc., Natick, Massachusetts, United States) was used for statistical analysis. The functional connectivity results were visualized using BrainNetViewer (v1.61) [65].

### Statistical analysis

All inclusion/exclusion criteria were pre-established, and no animals or samples were excluded in the analysis. Data were presented as means ± SEM of three or more independent experiments unless stated otherwise. All statistical analyses were performed using GraphPad Prism 8 software (GraphPad Software Inc., La Jolla, CA, USA). The behavior test score was compared using two-way analysis of variance (ANOVA) followed by Tukey’s post hoc test. Except for special instructions, the other experiments were analysis using t-test. *p* < 0.05 was considered statistically significant.

## Supplementary information

Supplementary materials

Original Data File

aj-checklist

## Data Availability

All data are available in the main text or the supplementary materials.
