# Supplementary material for: A small molecule 20C from Gastrodia elata inhibits α-synuclein aggregation and prevents progression of Parkinson’s disease
Source: Cell Death Dis. 2023 Sep 6;14(9):594. doi: 10.1038/s41419-023-06116-0 (PMC10482970; doi:10.1038/s41419-023-06116-0)
Supplement: Supplementary file 1 — Supplementary materials [file 41419_2023_6116_MOESM1_ESM.docx]

Supplemental Information

**A small molecule 20C from *Gastrodia elata* inhibits α-synuclein aggregation and prevents progression of Parkinson’s disease**

Ye Peng, Junrui Ye, Shasha Wang, Wenbin He, Zhongping Feng, Hongshuo Sun, Shifeng Chu, * Zhao Zhang, * Naihong Chen*

*Corresponding author. Email: [chushifeng@imm.ac.cn](mailto:chushifeng@imm.ac.cn); [zhangzhao@imm.ac.cn](mailto:zhangzhao@imm.ac.cn); [chennh@imm.ac.cn](mailto:chennh@imm.ac.cn)

**This PDF file includes:**

Figs. S1 to S10

Tables S1 to S3

Fig. S1.


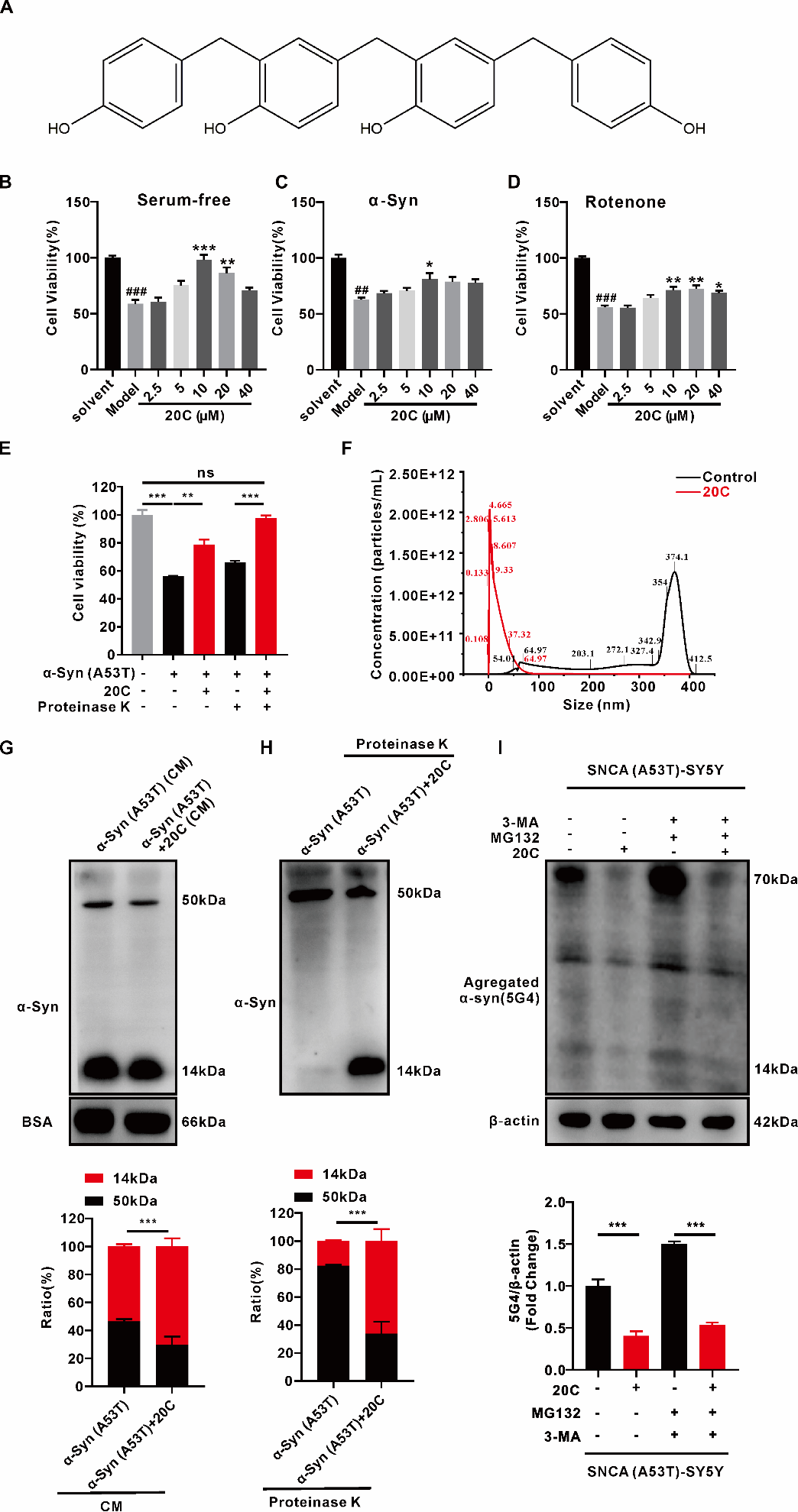


**Fig. S1. Determination of anti-PD activities of 20C *in vitro*.** (A) Chemical structure of 20C. (B) Protective effect of 20C on serum-free injury model. (C) Protective effect of 20C on α-Syn injury model. (D) Protective effect of 20C on rotenone injury model. (E) The presence of 20C reduces the toxicity of α-Syn oligomers. (F) Determination of the fibril size population in absence (black) and presence (red) of 20C after proteinase K digestion using nano ZS system. (G) Representative immunoblot and quantification analysis of α-syn protein levels in cell culture medium. (H) Proteinase K digestion of the α-Syn oligomers. (I) Representative immunoblot and quantification analysis of SNCA (A53T)-SY5Y from α-Syn (A53T), α-Syn (A53T)+20C, α-Syn (A53T)+MG132 (10 μM) +3-MA (5 mM), α-Syn (A53T) +20C+ MG132 (10 μM) +3-MA (5 mM) treatment. The experiments were analysis using one-way ANOVA followed by Tukey’s post hoc test. Error bars are represented as SEM of mean values. (*^###^p* < 0.01 vs. the Control group, ***p* < 0.01, ****p* < 0.001 *vs.* the Model group in B-D, ***p* < 0.01, ****p* < 0.001 between the two groups in E-H).

Fig. S2.


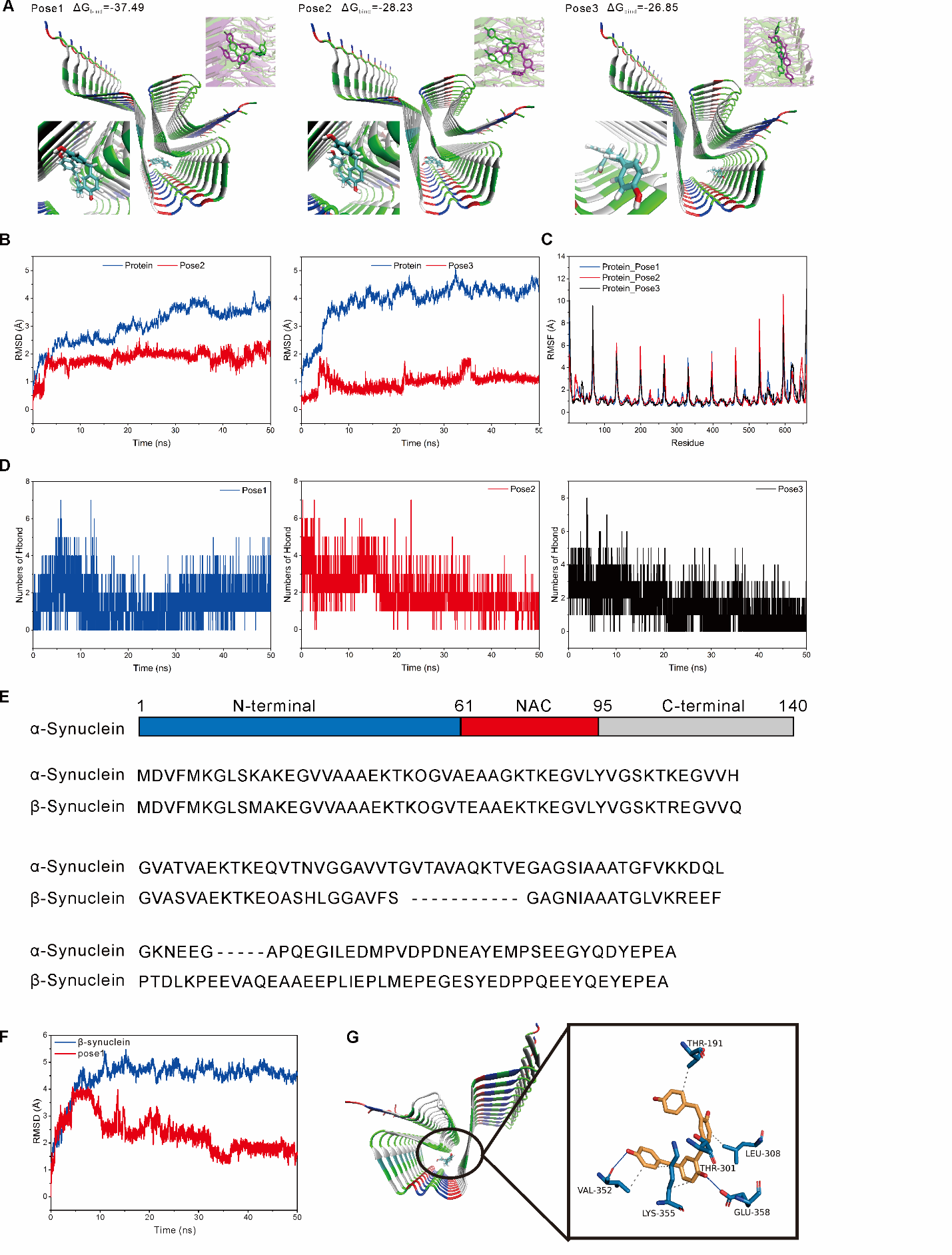


**Fig. S2. α-Syn fibrils accommodated 20C.** (A) Binding site predicted by the Discovery Studio software. Binding energies (∆G_bind_) are expressed in Kcal/mol. Composite diagram of the complexes before and after simulation (The top right corner, purple shows the conformation at the last moment of simulation, green shows the conformation before simulation). (B) RMSD results of pose 1, 2, 3 with time in the simulation process. (C) RMSF results of residues during 50ns MD. (D) The variation diagram of hydrogen bond number formed by each pose with time in the simulation process. (E) (Top) Domain organization of α-Syn. The N-terminus (blue), non-amyloid-β component (NAC) (red) and C-terminus (grey) are shown. Bottom: Residues in the α-and β-synucleins alignment of the members of the synuclein family generated through Clustal-Womega. (F) RMSD results of 20C-β-Synuclein complex with time in the simulation process. (G) The binding mode based on 20C-β-Synuclein interaction dynamics simulation, overall view (left) and local view (right). The yellow rod-like structure is 20C, the blue rod-like structure is a protein residue, the blue solid line represents hydrogen bonding, and the gray dotted line represents hydrophobicity.

Fig. S3.


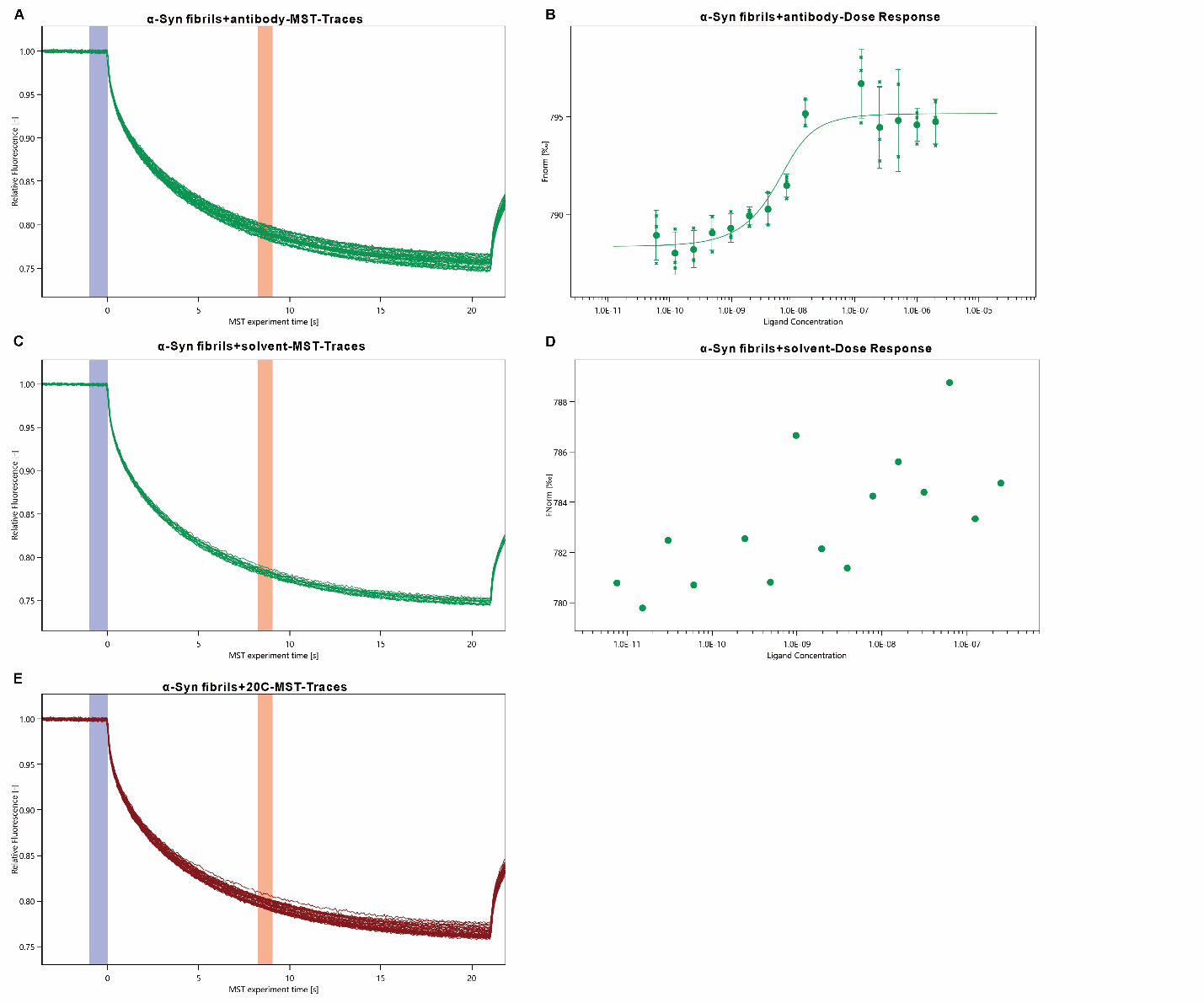


**Fig. S3. Measurement of binding affinity between α-synuclein fibril and 20C using MST.** (A) Fluorescence intensity versus time curve measurements of α-Syn fibrils binding with different concentrations of its antibody (Anti-Aggregated α-Synuclein 5G4, MABN389). (B) Dose-response curve and determination of Kd of antibody with α-Syn fibrils interaction. (C) Fluorescence intensity versus time curve measurements of α-Syn fibrils binding with solvent control experiment. (PBS containing 8% DMSO). (D) The binding curve could not be fitted in solvent control experiment. (E) Fluorescence intensity versus time curve measurements of α-Syn fibrils binding with different concentrations of 20C.

Fig. S4.


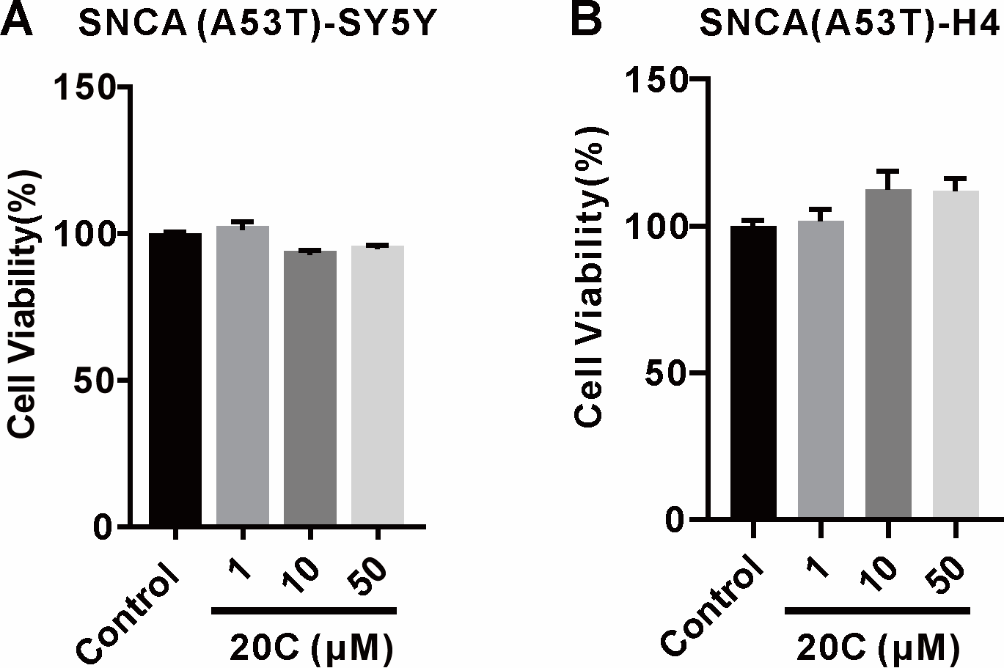


**Fig. S4. Potential toxicity of 20C for SNCA(A53T) overexpression neurons.** (A) The viability of SNCA (A53T)-SY5Y cells incubated with different concentrations of 20C. (B) The viability of SNCA (A53T)-H4 cells incubated with different concentrations of 20C.

Fig. S5.


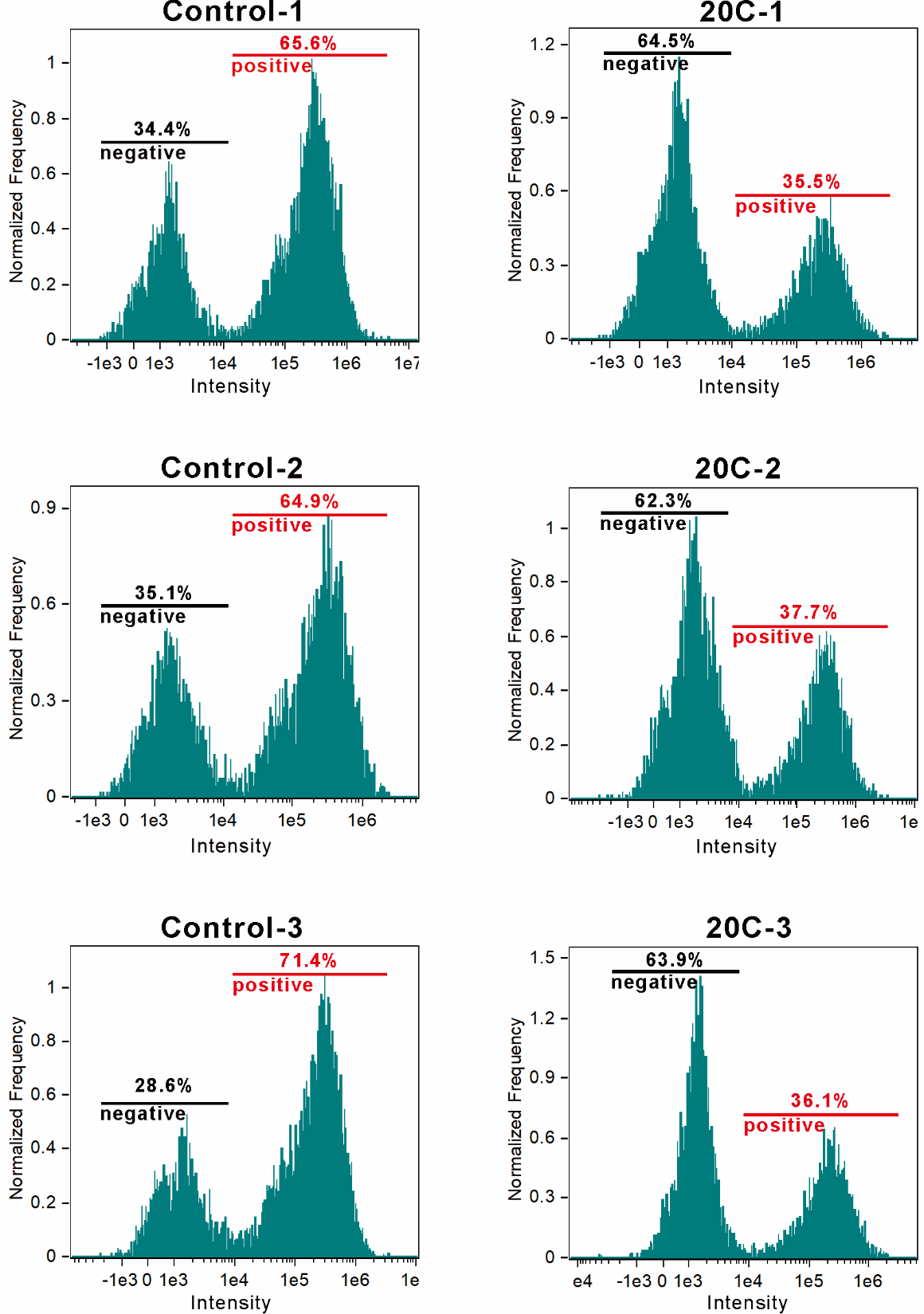


**Fig. S5. EGFP fluorescence intensity in SNCA (A53T)-H4 cells.**

Fig. S6.


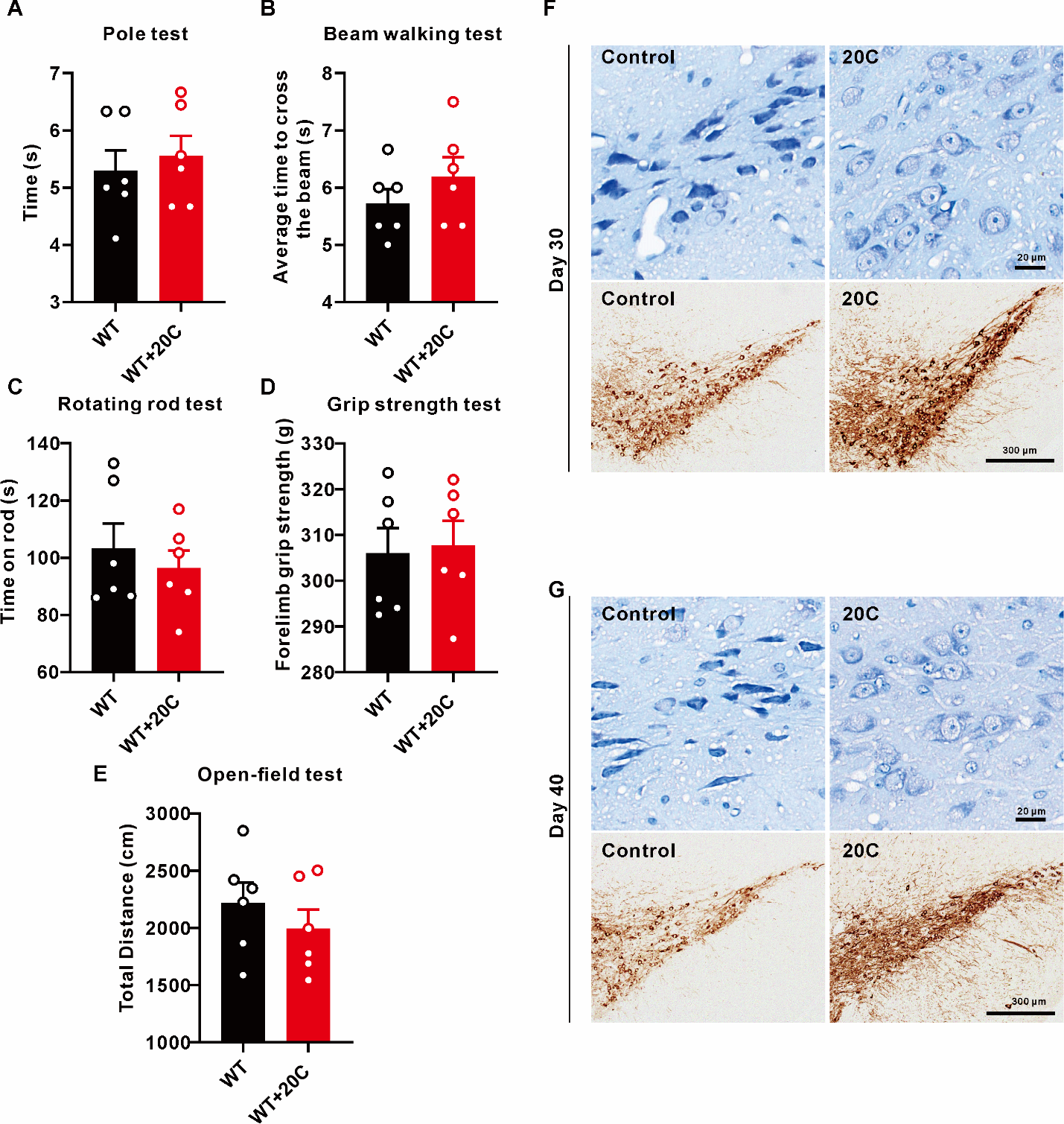


**Fig. S6. The behavioral performance in WT mice.** Time to descend pole (A), to traverse beam apparatus (B), to fall latency from an accelerating rotarod (C) and grip strength analysis (D). (E) Total distance of open field test. (F) The TH immunostaining and Nissl staining of SNc at Day 30. Scale bar=20 µm in Nissl staining and Scale bar=300 µm in TH immunostaining. (G) The TH immunostaining and Nissl staining of SNc at Day 40. Scale bar=20 µm in Nissl staining and Scale bar=300 µm in TH immunostaining.

Fig. S7.


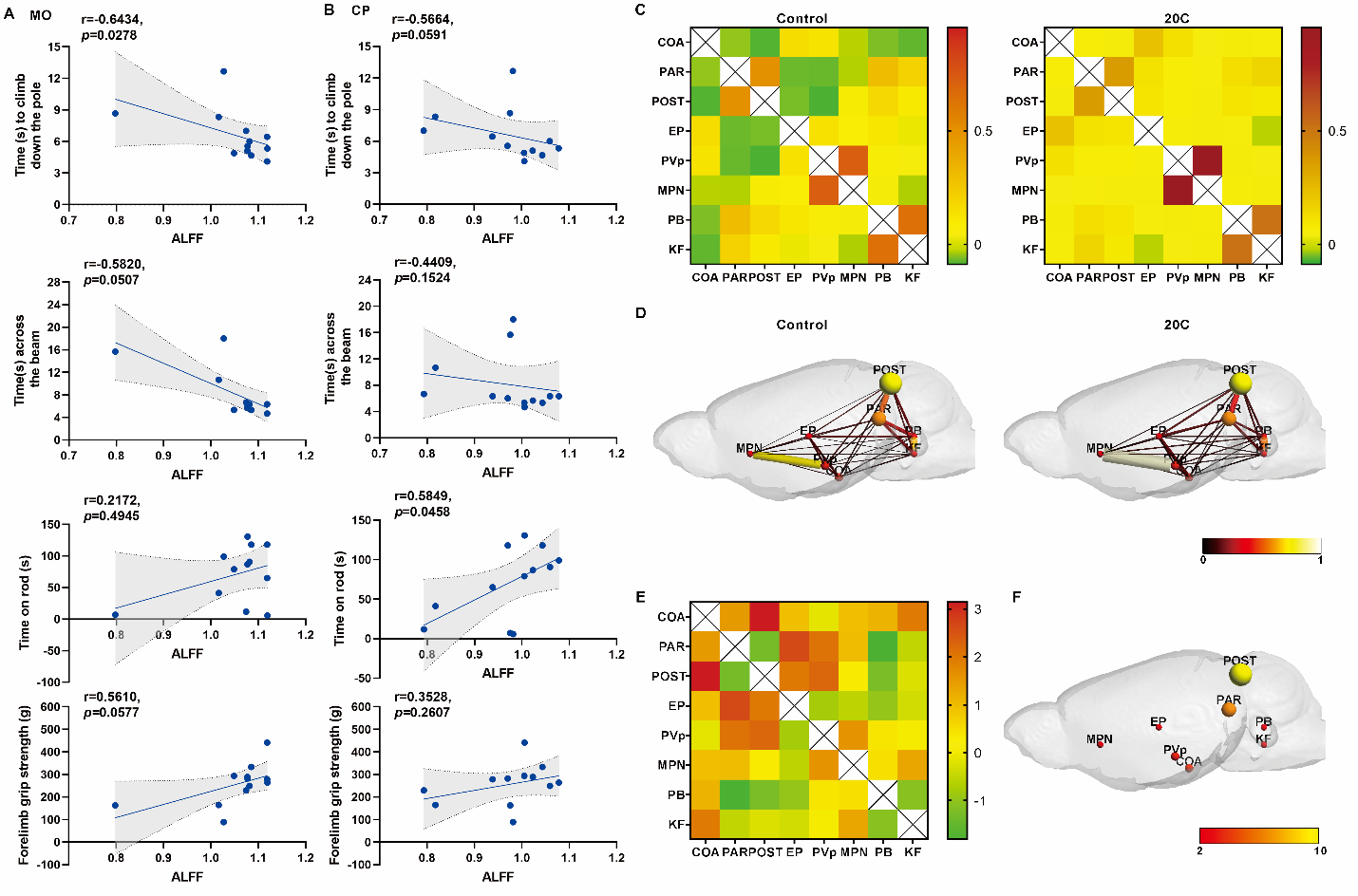


**Fig. S7. 20C improved functional connectivity in A53T α-Syn transgenic mice.** (A) the relationship between the assessed motor behaviors and the ALFF changes in MO induced by A53T and 20C. (B) the relationship between the assessed motor behaviors and the ALFF changes in CP induced by A53T and 20C. (C) Heatmap of the regions of interest (ROI) are connected in pairs. The control group mice show matrices of clearly lower Z-score (Left). Upon 40 days of 20C treatment (Right), the Z-score values of the transgenic animals significant increased. (D) In this schematic the functional connectivity between pairs of nodes is marked by lines of increasing thickness with increasing Z-score values, indicating increasing functional connectivity. The intra-node strength is depicted by larger or smaller spheres. (E) Brain regions with significant differences are indicated by different colors according to T-values. (F) The intra-node strength is depicted by larger or smaller spheres.

Fig. S8.


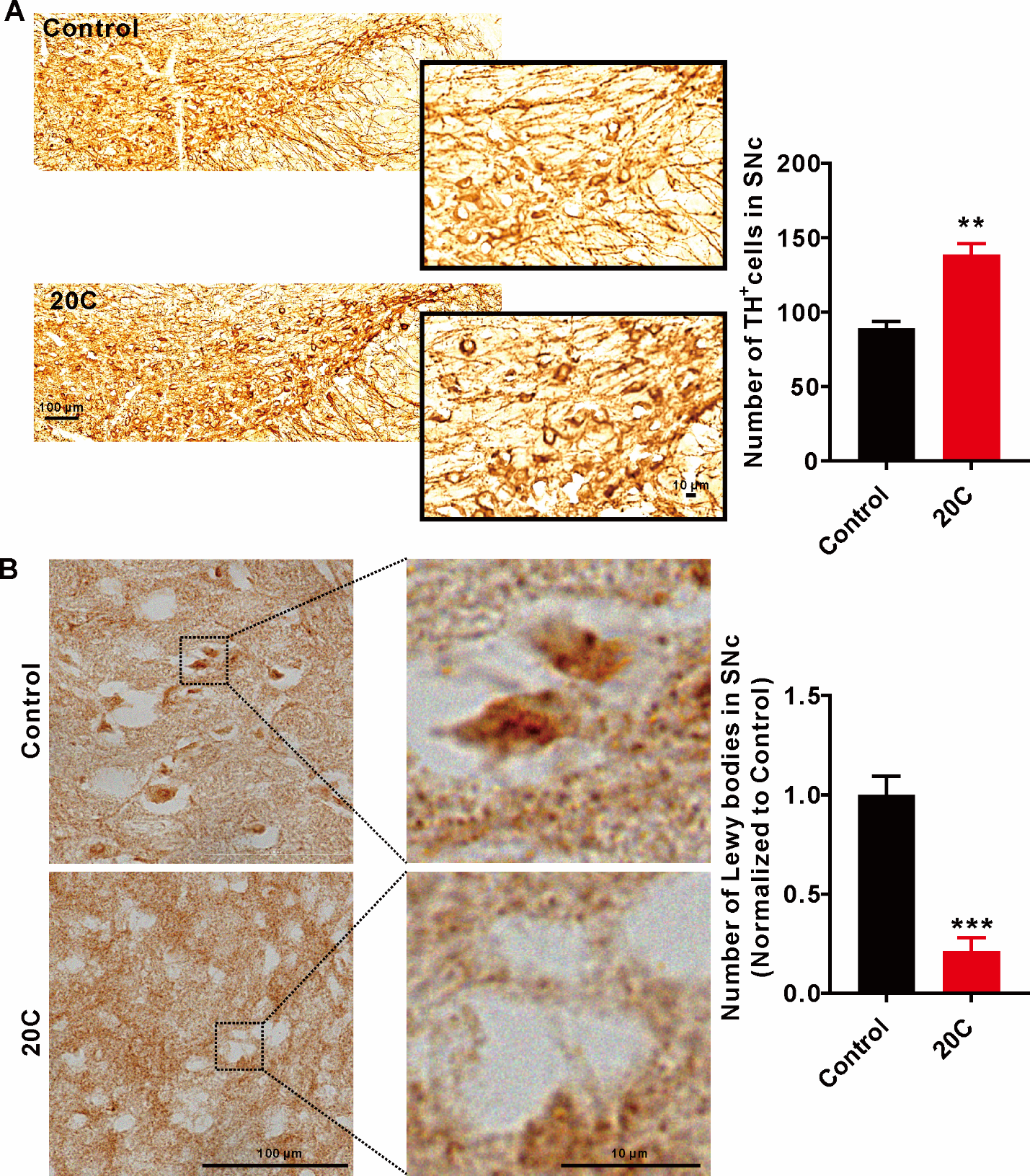


**Fig. S8. 20C could protect the terminal of DA neurons in the SNc.** (A) Representative image and quantification analysis of TH immunostaining of SNc. Scale bar=100 µm. Enlarge figure=10 µm. (B) Representative image and quantification analysis of Lewy bodies of SNc. Scale bar=100 µm. Enlarge figure=10 µm. Error bars are represented as SEM of mean values, n=3. ***p* < 0.01, ****p* < 0.001 *vs.* control group.

Fig. S9.


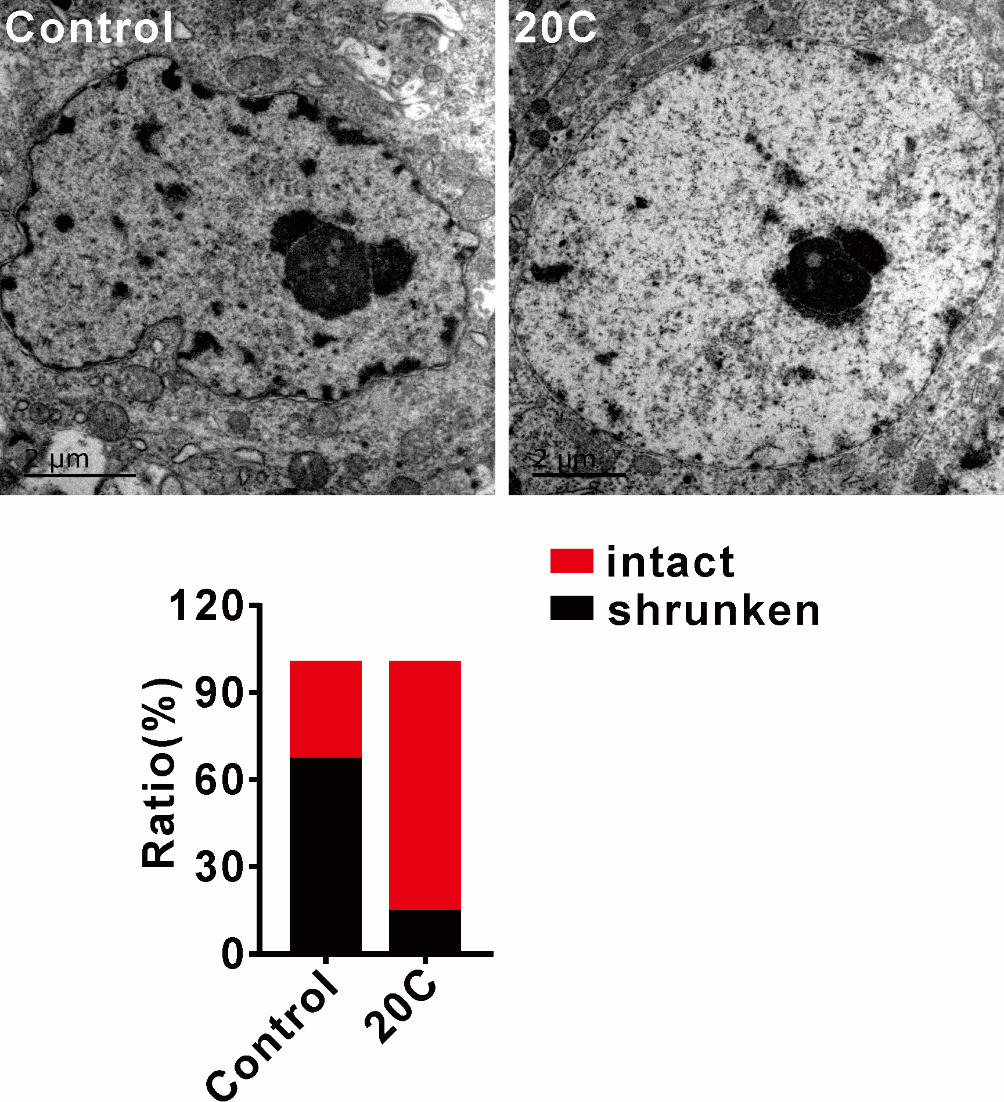


**Fig. S9. Representative electron microscopy photomicrographs of neuronic nuclear in SNc.** Scale bar=2 µm. Percentage of nuclear content in both states (intact and shrunken).

Fig. S10.


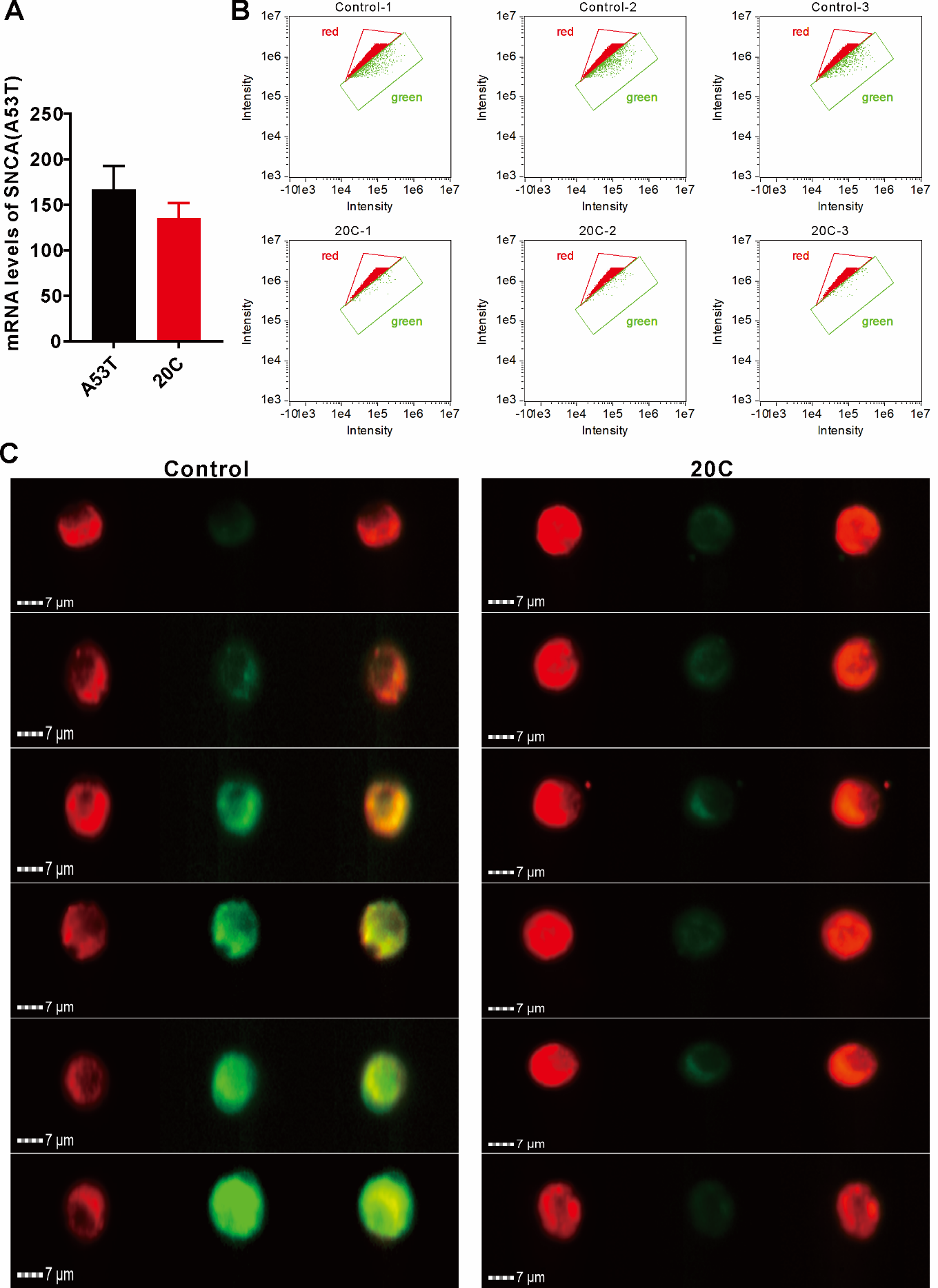


**Fig. S10.** Mitochondrial membrane potential was stained with JC-1, and then assessed using imaging flow cytometry.

Tab. S1. Binding free energies and energy components predicted by MM/GBSA (kcal/mol).

| **System name** | | **Protein/Pose1** | **Protein/Pose2** | **Protein/Pose3** |
| --- | --- | --- | --- | --- |
| **ΔE_vdw_** | | -47.70±1.73 | -35.02±2.82 | -45.86±1.77 |
| **ΔE_elec_** | -27.67±3.44 | | -2.33±7.96 | -17.04±6.64 |
| **ΔG_GB_** | 44.57±2.22 | | 17.79 ±3.29 | 43.37 ±8.31 |
| **ΔG_SA_** | -6.68±0.30 | | -4.45±0.06 | -7.32±0.23 |
| **ΔG_bind_** | -37.49±1.69 | | -28.23±3.07 | -26.85±2.53 |

ΔE_vdW_: van der Waals energy.

ΔE_elec_: electrostatic energy.

ΔG_GB_: electrostatic contribution to solvation.

ΔG_SA_: non-polar contribution to solvation.

ΔG_bind_: binding free energy.

Tab. S2. Antibodies, chemicals, assay kits, and instrument used in this study.

| **Reagent or resource** | **Source** |
| --- | --- |
| **Protein** |  |
| Alpha-synuclein (mutated A53T) protein (ab256150) | Abcam |
| **Antibodies** |  |
| TH (SC25269, 1:500) | Santa cruz |
| TH (AB152, 1:500) | Sigma |
| Syn 211 (32-8100, 1: 200) | ThermoFisher |
| α-Syn phosphorylation S129 (ab51253, 1: 1000) | Abcam |
| α-Syn 5G4 (MABN389, 1: 200) | Sigma |
| Hochest33342 (1: 1000) | Heychem |
| MFN1(13798-1-AP, 1: 1000) | ThermoFisher |
| MFN2 (12186-1-AP, 1: 1000) | ThermoFisher |
| p-Drp1 (Ser-637) (DF2980, 1: 1000) | Affinity |
| p-Drp1 (Ser-616) (AF8470, 1: 1000) | Affinity |
| Drp1 (SC32898, 1: 1000) | Santa cruz |
| BSA (K106448P, 1: 1000) | Solarbio |
| **Other Materials** |  |
| Rat pheochromocytoma cells (PC12) | Institute of Materia Medica, Chinese Academy of Medical Sciences and Peking Union Medical College |
| Human neuroblastoma cells (SH-SY5Y) | Institute of Materia Medica, Chinese Academy of Medical Sciences and Peking Union Medical College |
| Human neuroglioma cells (H4) | Shanghai Yaji Biological Company |
| The cell culture regents | Life-Technologies-Gibco |
| 3-(4,5-dimethylthiazol-2-yl)-2,5-diphe-nyltetrazolium bromide (MTT, M6494) | Thermo Fisher Scientific |
| Carbon-coated copper grid, 230 mesh | Zhongjingkeyi Technology |
| Uranium acetate solution | Zhongjingkeyi Technology |
| Thioflavin-T (T3516) | sigma |
| TRIzol reagent | TransGen Biotech |
| TransScript one-step gDNA removel and cDNA synthesis Super MiX | TransGen Biotech |
| SYBR Green PCR system | TransGen Biotech |
| Mito-tracker staining (1999441) | Invitrogen |
| Reactive oxygen species (ROS) assay kit (S0033S) | Beyotime |
| Mitochondrial membrane potential assay kit with JC-1 (C2006) | Beyotime |
| Cell Mito Stress Test Kit (103015-100) | Agilent Technologies |
| 3-Methyladenine (3-MA) (S2767) | Selleck |
| MG-132 | Beyotime |
| **Instrument** |  |
| EnSpire Multimode Reader | PerkinElmer |
| Zetasizer Nano ZS | Malvern Instruments Ltd |
| Monolith NT.115 instrument | Nano-Temper Technologies |
| Grip testing equipment, YLS-13A | Xuzhou Lihua Electronic Technology |
| imaging fow cytometer, IN Cell Analyzer 1000 | GE |
| NanoPhotometer N50 Touch | IMPLEN |
| Nanodrop 2000 | Thermo Scientific |
| Seahorse XF24 analyzer | Agilent Technologies |

Tab. S3. The sequences of the primers used for real-time qPCR.

| **Gene** | **Primer sequences** |
| --- | --- |
| Dnm1l | AACAGGCAACTGGAGAGGAA |
|  | GCAACTGGAACTGGCACAT |
| Mfn1 | CCAGGTACAGATGTCACCACAG |
|  | TTGGAGAGCCGCTCATTCACCT |
| Mfn2 | GTGGAATACGCCAGTGAGAAGC |
|  | CAACTTGCTGGCACAGATGAGC |
| Naxe | GAGCCATTCCACAGCATCCTGA |
|  | GAGTAAGTCTGGTTGGATTCCGC |
| Slc25a16 | GACAGCGGTTATCTGCACTTACC |
|  | GCATCAGACCTCGGTAGAATCC |
| Mrps24 | ACTACATCGCACACCGAAAGGG |
|  | CAGAACAATCTGGTCAGCCAGG |
| Sdhaf1 | AACCGACGTGCTGCGTATCGAG |
|  | TGTCGCGTCGCCGGGCTCTT |
| Cox11 | GGAACAAGACGGTGCTCACCTA |
|  | CACTGCTGATCCTCCAAGTCCA |
| Fdps | GGTGGTTCAGTGTCTGCTACGA |
|  | CGCCTCATACAGTGCTTTCACC |
| Comtd1 | CCTTCGCTACTGGTCTCTTGCT |
|  | ATGGAGCGGCTCAGCAGATACT |
| Slc25a51 | AAGACGACCACGCTGGCACTTA |
|  | TGGAGTGAGAATGGCTTCCGTC |
| Endog | CCACACCTCAACCAGAATGCCT |
|  | CACATAGGACTTCCCATCAGCC |
| Prss35 | ACTGTGTCCACGATGGGAAGGA |
|  | GCTCCTTTTGGAACCTCTGCGT |
| Actb | GCAGGAGTACGATGAGTCCG |
|  | ACGCAGCTCAGTAACAGTCC |
