# Supplementary material for: A small molecule 20C from Gastrodia elata inhibits α-synuclein aggregation and prevents progression of Parkinson’s disease
Source: Cell Death Dis. 2023 Sep 6;14(9):594. doi: 10.1038/s41419-023-06116-0 (PMC10482970; doi:10.1038/s41419-023-06116-0)
Supplement: Supplementary file 2 — Original Data File [file 41419_2023_6116_MOESM2_ESM.docx]

**Original western blots**

**A small molecule 20C from Gastrodia elata inhibits α-synuclein aggregation and prevents progression of Parkinson’s disease**

Ye Peng, Junrui Ye, Shasha Wang, Wenbin He, Zhongping Feng, Hongshuo Sun, Shifeng Chu, * Zhao Zhang, * Naihong Chen*

*Corresponding author. Email: [chushifeng@imm.ac.cn](mailto:chushifeng@imm.ac.cn); [zhangzhao@imm.ac.cn](mailto:zhangzhao@imm.ac.cn); [chennh@imm.ac.cn](mailto:chennh@imm.ac.cn)

**Fig. 5E**

Substantia nigra (TH)


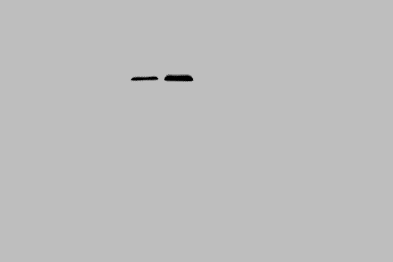

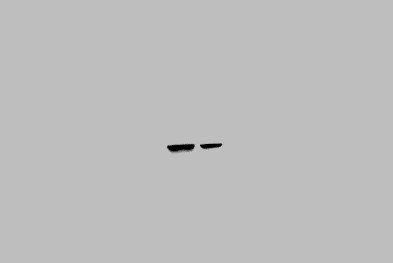

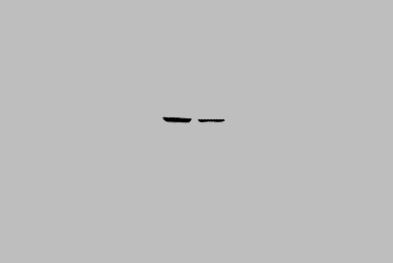


60kDa

60kDa

60kDa

Substantia nigra (β-actin)


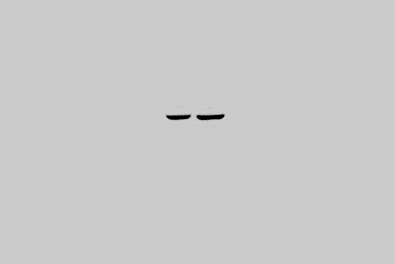

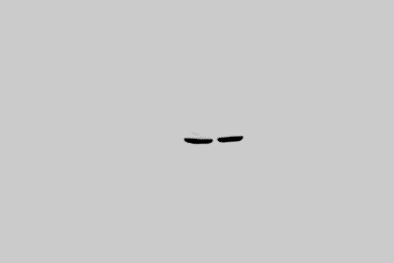

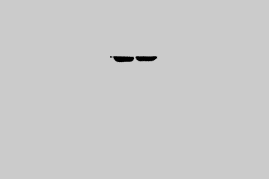


42kDa

42kDa

42kDa

Striatum (TH)


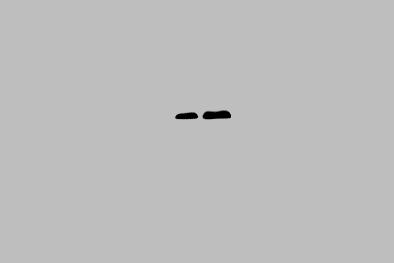

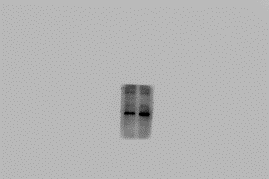

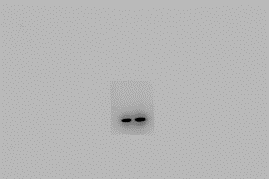


60kDa

60kDa

60kDa

Striatum (β-actin)


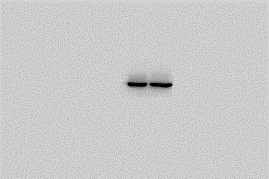

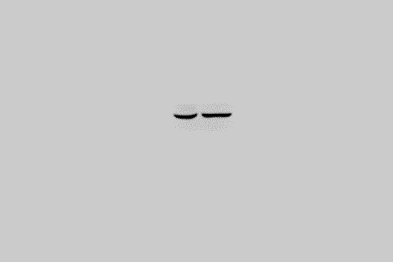

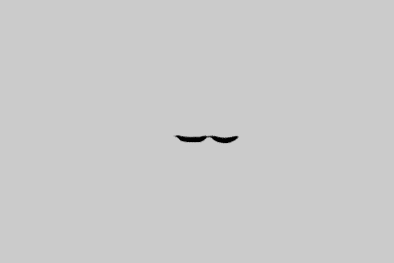


42kDa

42kDa

42kDa

**Fig. 5N**

α-Syn


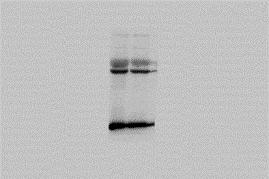

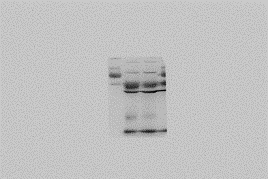




14kDa

14kDa

14kDa

β-actin


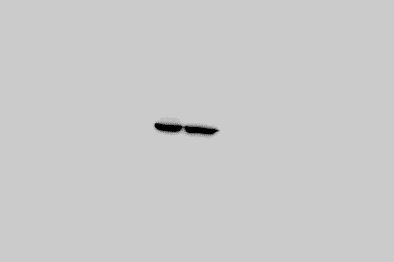



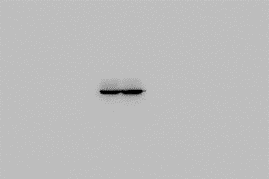


42kDa

42kDa

42kDa

Aggregated α-Syn


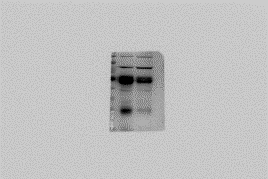

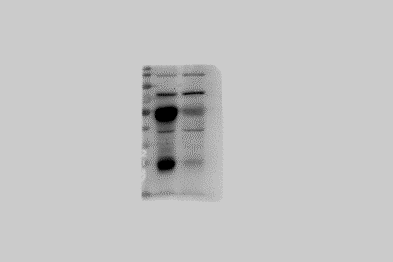




70kDa

70kDa

70kDa

β-actin




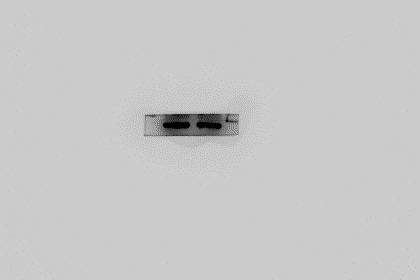




42kDa

42kDa

42kDa

Nitrated α-Syn









70kDa

70kDa

70kDa

β-actin


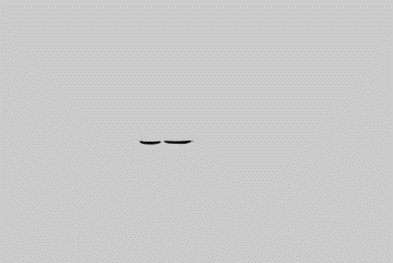






42kDa

42kDa

42kDa

Phosphorylated α-Syn (S129)









70kDa

70kDa

70kDa

β-actin









42kDa

42kDa

42kDa

**Fig. 6G**

P-DRP1-S616


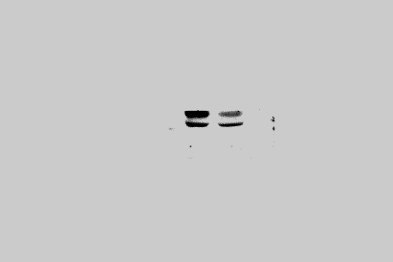

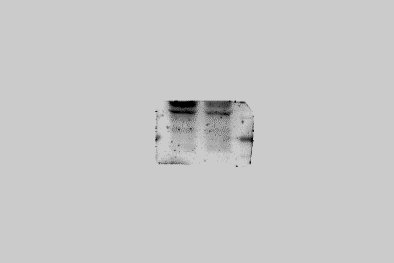

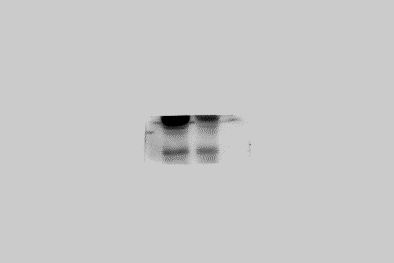


80kDa

80kDa

80kDa

P-DRP1-S637


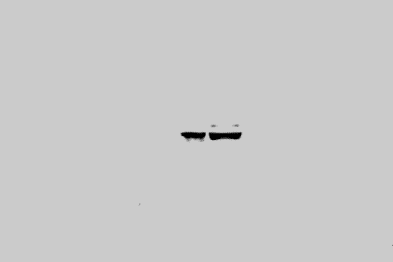

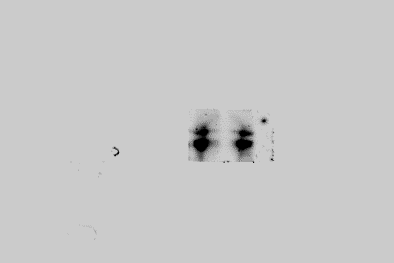

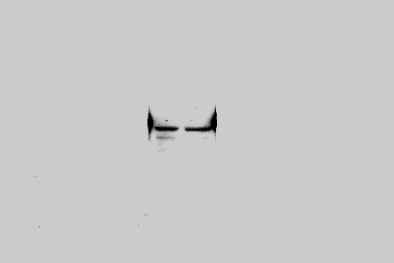


80kDa

80kDa

80kDa

DRP 1


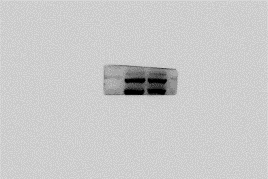

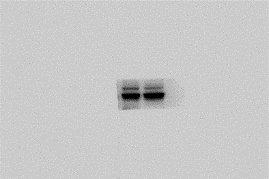

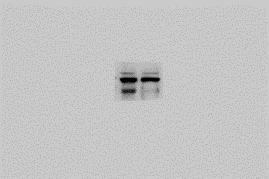


80kDa

80kDa

80kDa

MFN1


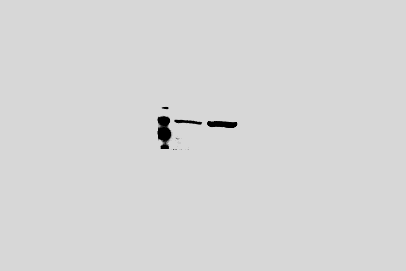

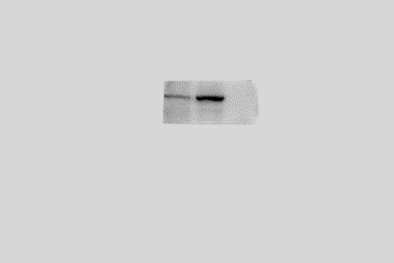

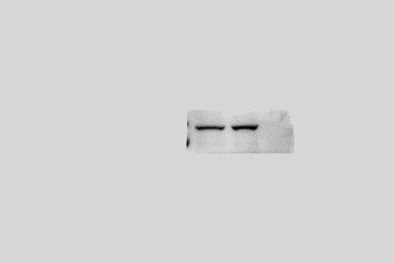


90kDa

90kDa

90kDa

MFN2


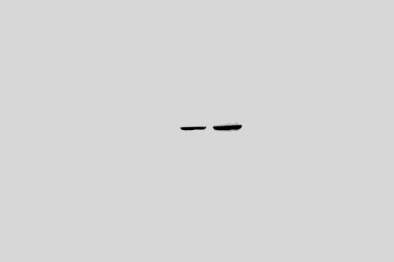

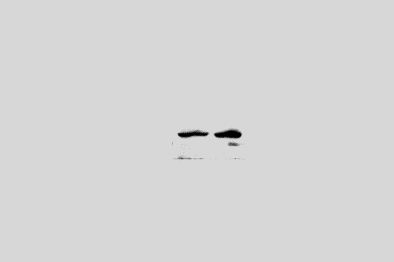

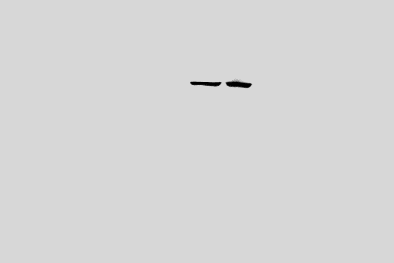


86kDa

86kDa

86kDa

MIRO1


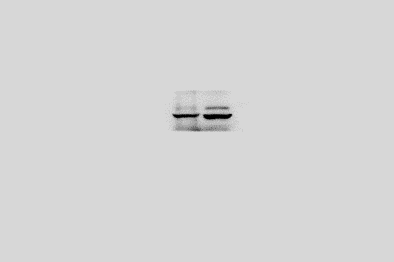



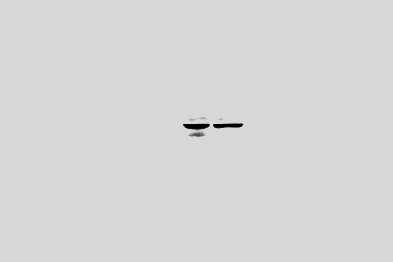


71kDa

71kDa

71kDa

β-actin


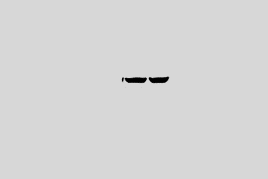

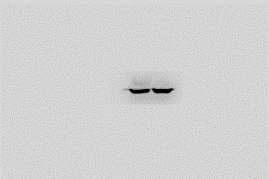

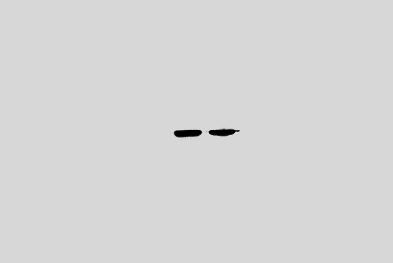


42kDa

42kDa

42kDa

**Fig. S1G**

α-Syn




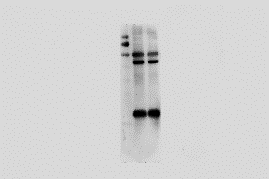

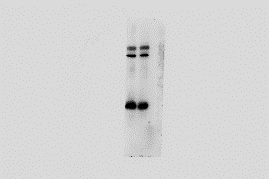


14kDa

14kDa

14kDa

50kDa

50kDa

50kDa

β-actin









42kDa

42kDa

42kDa

**Fig. S1H**

α-Syn


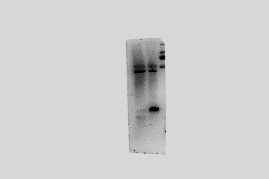

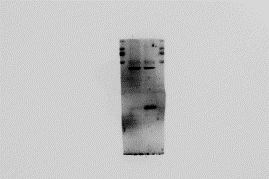




50kDa

14kDa

14kDa

14kDa

50kDa

50kDa

**Fig. S1I**

Aggregated α-Syn


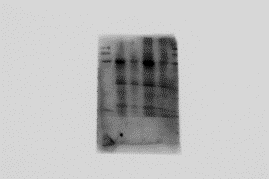

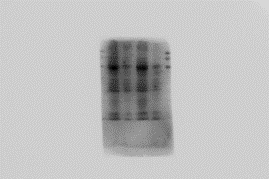

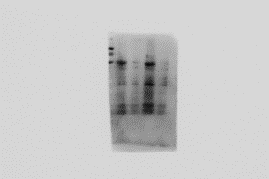


70kDa

70kDa

70kDa

β-actin









42kDa

42kDa

42kDa
